# Changes in Reef Fish Community Structure Following the Deepwater Horizon Oil Spill

**DOI:** 10.1038/s41598-020-62574-y

**Published:** 2020-04-09

**Authors:** Justin P. Lewis, Joseph H. Tarnecki, Steven B. Garner, David D. Chagaris, William F. Patterson

**Affiliations:** 1 University of Florida, Fisheries and Aquatic Sciences, Gainesville, Florida 32608 USA; 2 University of Florida, Nature Coast Biological Station, Cedar Key, FL 32625 USA

**Keywords:** Environmental impact, Community ecology

## Abstract

Large-scale anthropogenic disturbances can have direct and indirect effects on marine communities, with direct effects often taking the form of widespread injury or mortality and indirect effects manifesting as changes in food web structure. Here, we report a time series that captures both direct and indirect effects of the *Deepwater Horizon* Oil Spill (DWH) on northern Gulf of Mexico (nGoM) reef fish communities. We observed significant changes in community structure immediately following the DWH, with a 38% decline in species richness and 26% decline in Shannon-Weiner diversity. Initial shifts were driven by widespread declines across a range of trophic guilds, with subsequent recovery unevenly distributed among guilds and taxa. For example, densities of small demersal invertivores, small demersal browsers, generalist carnivores, and piscivores remained persistently low with little indication of recovery seven years after the DWH. Initial declines among these guilds occurred prior to the arrival of the now-widespread, invasive lionfish (*Pterois* spp.), but their lack of recovery suggests lionfish predation may be affecting recovery. Factors affecting persistently low densities of generalist carnivores and piscivores are not well understood but warrant further study given the myriad ecosystem services provided by nGoM reef fishes.

## Introduction

The nature, frequency, and intensity of disturbance are important drivers of community structure^1–3^, and it is well established that evolutionary history^4^, historical disturbance regimes^2,4^, and the prior state of a community^2,5,6^ affect its response^7^. Although natural disturbances can be important for maintaining diverse, resilient species assemblages^8^, research focused on the impacts of chronic anthropogenic stressors on biodiversity has revealed that even specious communities, presumed to be resilient, can respond unpredictably to natural and anthropogenic disturbances^5,9,10^. This is particularly true in marine systems which have experienced impacts from human activities for centuries and are severely degraded as a result^11,12^. Numerous examples exist of long-term community shifts from estuarine^13^, coral reef^14,15^, and continental shelf^16,17^ systems, and it is not uncommon for communities to remain unaffected by localized or moderate disturbances^5^, only to exhibit a non-linear response following a series of disturbances^18^ or a single event of sufficient scale or intensity^19^.

The 2010 *Deepwater Horizon* Oil Spill (DWH) was the epitome of a large-scale, anthropogenic disturbance capable of producing substantial community-level impacts. Over an 87-day period, approximately 4.9 million gallons of oil^20^ was released into northern Gulf of Mexico (nGoM) at a depth of ~1,500 m producing a surface slick of ~40,000 km^2^ at its maximum extent^21^. Between 4 and 14% of the total discharge was transported to the benthos by contaminated marine snow^21–24^, thus exposing numerous pelagic and benthic communities to toxic polycyclic aromatic hydrocarbons (PAHs)^25^ as well as emulsifying dispersants^26^. Both lethal and sublethal effects (e.g., compromised immune^27^ and endocrine function^28^, developmental abnormalities^29^, reduced growth^30^, and impaired olfaction^31^ and predator avoidance^32,33^) of oil exposure have been well documented for numerous taxa^34–37^, and negative effects at the organismal level had the clear potential to elicit effects on community structure through bottom-up^38^ or top-down mechanisms^39,40^.

Much of the effort to document the community-level responses to the DWH was focused on monitoring coastal habitats that provide critical nurseries for several marine taxa^41^, are widely studied by community ecologists^42^, and whose proximity favored the rapid collection of critical baseline data^43^. Despite extensive shoreline oiling^44^, impacts were mostly relegated to heavily oiled, coastal sites in Louisiana where significant vegetation loss occurred along the marsh edge^45^. Inshore communities, particularly nekton and fish assemblages, in areas of limited exposure showed little indication of negative effects higher than the organismal level^46–49^. Multiple assessments of community-level effects on the nGoM continental shelf also indicated no impacts or a resilient recovery. For example, changes in plankton communities were relatively brief and isolated to the active spill period^50–52^; post-DWH meiofauna diversity and abundance were comparable to pre-spill estimates^53^; and macroinfauna abundance and diversity showed no signs of impacts^54^.

The lack of discernable DWH effects on community structure have been documented repeatedly but mostly for communities dominated by short-lived taxa^45,55,56^ more likely to be resilient to such a distrubance^57^. Considerably less is known about the impact to and response of neritic fish communities, including reef fish assemblages in the nGoM. These fish communities are diverse^58,59^, including numerous short-lived, small demersal species, as well as long-lived, fisheries species likely to be resistant to additional sources of mortality^60,61^. Key to the persistence of many fisheries species is the maintenance of multiple year classes of relatively old, mature individuals (i.e., the storage effect^60^) that experience low natural mortality, which minimizes biomass loss between periodically strong year classes^61^. Thus, compensatory population increases following DWH-induced mortality would likely be slow.

Resistance is also conferred through their high vagility and generalist diets which would allow individuals to disperse to avoid acute mortality^62^ while exploiting locally abundant prey^63,64^. However, evidence of sub-lethal PAH exposure suggests negative effects reached well beyond the footprint of the surface slick^37,65^, and recent ecosystem simulations suggest mortality resulting from acute resource limitation was perhaps more severe and widespread than exposure mortality^66^. Indeed, changes in feeding ecology, trophic position, and condition of red snapper (*Lutjanus campechanus*) were documented following the DWH^63,67^ and provide empirical support for a shift in resource availability with possible negative effects on population productivity^67^. Similar shifts in trophic position and pathways were observed in other reef fish species concomitant with petrocarbon cycling through the nGoM food web^68,69^. However, to date few data have been presented linking declines in reef fish abundance or community shifts that followed either direct effects of the DWH or indirect effects via food web impacts^70^.

The lack of information regarding community-level responses following the DWH may stem, in part, from the lack of pre-spill baseline data for many taxa and communities. Here, we analyze a time-series of nGoM reef fish community data that span an eight-year period starting before the DWH. We test whether significant changes occurred in reef fish community structure following the DWH, and if so whether changes occurred evenly across trophic guilds or were more concentrated within specific guilds. Results are presented in the context of acute, direct and chronic, indirect effects of the DWH, as well as factors that may affect the resiliency of reef fish communities.

## Methods

### Site description and data collection

**Figure 1:**
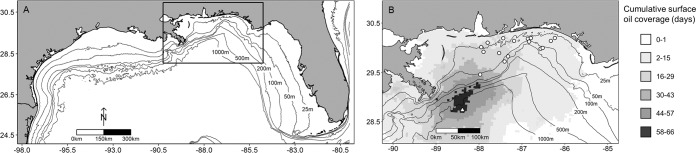
Map of the northern Gulf of Mexico (**A**) and sampling region (**B**). Panel B shows the location of the Deepwater Horizon blowout at the Mississippi Canyon-252 wellhead (triangle), and the natural reefs surveyed from 2009 to 2010 (circles). The shaded area represents the cumulative surface oil coverage in days. Maps were produced in R version 3.5.1^77^.

Fish communities were surveyed at 16 natural reefs (Fig. 1) in the nGoM with a VideoRay Pro4 mini remotely operated vehicle (ROV) during 2009–2017. Reefs were randomly selected from a series of sites surveyed in 2008–2009^71^, encompassed a depth range of 17–72 m, and were distributed over an 8,000 km2 area of the continental shelf. Sites were representative of the morphologically variable hard bottom habitat in the region and included low relief ledges, rocky ridges, rock rubble mounds, and flat limestone block reefs^72^. The epibenthic communities are dominated by coralline algae, soft corals (e.g., black corals, gorgonians, and octocorals), and sponges with limited coverage by azoozanthellate, ahermatypic corals^72^. Although impacts to epibenthic species were observed west of our sampling region^73^, sedimentation of contaminated marine snow was patchy^24^, and we observed no signs of habitat degradation (e.g., oiling or injured/deceased coral colonies). Thus, monitoring of the epibenthic community was not undertaken.

In total, 250 ROV surveys were completed between 2009 and 2017. The pre-DWH portion of our time series consisted of 26 surveys across 11 sites and occurred from the summer of 2009 to the spring of 2010. All 2010 surveys classified as pre-DWH occurred prior to surface oil entering our sampling region; surveys grouped in the 2010 time bin were sampled in November. During 2011–2013, sites were typically surveyed in the spring, summer, and fall, but later in the time series funding constraints limited sampling to summer months. Each ROV survey consisted of 3 to 4 orthogonal, 25-m transects either 1 or 2 m off the bottom, depending on visibility^70,71^. The ROV was equipped with a 570-line color camera with wide-angle (116°) lens for real-time viewing, twin forward projecting LEDs (3,600 lumens), and rated to a depth of 170 m. An additional forward-facing, high definition (1080p at 60–120 fps) camera (GoPro Hero 2, 3, or 4) was mounted at a 45° angle to the ROV’s float block above the internal camera to record high definition video of the reef fish community.

Videoo samples were analyzed on a high-resolution monitor; observed fishes were identified to the lowest taxonomic level possible and enumerated. Counts were summed across transects to estimate the total abundance for each site. For active, schooling species prone to double counting (e.g., scads, herrings, etc.), the total abundance across all transects was superseded by an estimate of the minimum number of individuals within a school obtained while maneuvering between transect locations. Species abundances were converted to densities by dividing by the total area sampled following the methods of Patterson *et al*.^71^. Results presented below only include those taxa identified to the level of species with the exception of purple reeffish (*Chromis scotti*) and dusky damselfish (*Stegastes fuscus*), which are difficult to distinguish on video footage and therefore combined into a single group, damselfish.

### Community analysis

Permutational multivariate analysis of variance (PERMANOVA) models were computed in PRIMER (v7) to test for temporal changes in reef fish community structure following the DWH. To reduce the influence of abundant species, taxa-specific densities were log(x + 1) transformed. A resemblance matrix was then computed based on the Bray-Curtis dissimilarity, with the inclusion of a dummy species at a density of 1. The dummy species was included because Bray-Curtis can behave erratically if few species are shared between sites^74^, which is important to consider when evaluating environmental impacts on community structure. The PERMANOVA model had a three-factor hierarchical design with site nested within month nested within year. The nested factors, site and month, were treated as random factors while year was treated as a fixed factor. Our model also included two covariates, depth and longitude, which were z-score transformed. The reasons for this approach were: (1) each covariate represents a gradient along which reef fish community structure naturally varies^58,75^; (2) cluster analysis of pre-DWH community structure did not identify groups that might justify the use of discrete categorical factors to evaluate depth or longitude effects; (3) the use of covariates as opposed to fixed factors permitted the inclusion of the entire data set; and, (4) at this scale there is not a clear relationship between straight line distance from the well head and impacts. Changes in community structure were also evaluated using common community indices of species richness (*S*), diversity (Shannon-Weiner *H*′), and evenness (Pielou’s *J*′). All indices were calculated using the *vegan* package^76^ in R^77^. Temporal changes were evaluated with linear mixed effects models (LMMs) using the *lme4* package^78^ followed by Dunnett’s multiple comparisons using the *multcomp* package^79^.

### Trophic guild and species-specific trends

We evaluated temporal changes in density for nine trophic guilds: herbivores, small demersal browsers, large demersal browsers, small demersal invertivores, large demersal invertivores, generalist carnivores, piscivores, reef planktivores, and pelagic planktivores, with small versus large indicating species generally smaller versus larger than 200 mm total length. Species were assigned to guilds based on dietary data, both from the literature (see Appendix A) and recent analyses, and densities were summed by guild for each ROV sample. In the case of small demersal invertivores, we excluded tomtate (*Haemulon flavolineatum*) from guild-level estimates because densities of this schooling grunt (Haemulidae) were highly variable and often an order of magnitude larger than other guild members. Their inclusion obscured the more general, guild-level pattern (Fig. S1). The species-specific analyses included taxa (n = 52) whose relative frequency of occurrence was >5% before or after DWH and for which sufficient data were available for model convergence.

Temporal trends in trophic guilds and species were assessed by computing standardized density indices with generalized linear mixed effects models (GLMM) following the delta approach^80,81^. This approach consists of two models, one to model the probability of observing zero individuals (hereafter, presence/absence) and a second to model the density given a guild or species was observed^81^. The product of the two sub-models was then used as the standardized density index for each guild or taxon. GLMMs included year as a factor and the repeated measures design was specified by including a random intercept parameter for each site. Longitude and depth were also included as covariates following z-score transformation. Least-squares means were calculated for each sub-model as the annual average from a reference grid of predictions across factor levels (i.e., years). Monte Carlo simulations were computed to estimate an annual density index and confidence intervals following the methods in Chagaris *et al*.^82^. Briefly, the product of the least-squares mean standard error and 10,000 random normal deviates X ∼ N(μ = 0, σ = 1) was added to the least-squares mean estimate of annual density. Error deviates of the log-normal model were adjusted when the log-normal and binomial least-squares mean were correlated (Pearson’s correlation p-value ≤ 0.05). Values of each Monte Carlo simulation were then back-transformed into their original measurement units to obtain a distribution of density values.

The results from binomial and log-normal models were also evaluated separately to infer whether temporal differences resulted from a significant change in presence/absence or non-zero abundance. Multiple comparisons performed using Dunnett’s method, as described above. For guilds and species observed prior to the DWH, comparisons were made between the pre- and post-DWH time periods with non-zero density estimates. For species only observed during the post-DWH time period, comparisons were made between the first and subsequent years with non-zero density estimates.

## Results

### Species composition

Our ROV dataset included 138 species from 43 families. The highest densities were observed for grunts and snappers (Lutjanidae) reflecting the fact either tomtate or vermilion snapper (*Rhomboplites aurorubens*) was the most abundant species in a given year (standardized density range 27–136 and 26–195 individuals 1000 km^2^, respectively) (Supplemental Table S1). Approximately 43% percent of reef fish species were distributed among five other families: Serranidae (15.2%), Carangidae (7.2%), Sciaenidae (5.8%), Sparidae (5.8%), and Pomacentridae (5.1%).

### Community analysis

PERMANOVA results indicated community structure significantly differed among years (Table 1). Both covariates and the random effect of month within year were also statistically significant, while the random effect of site within month within year was not significant. Of the eight pairwise comparisons between pre- and post-DWH periods, significant differences in community structure were observed in 2010–2011 and 2013–2017 (Table 2). Pairwise comparisons from the post-DWH portion of the time series (i.e., 2010–2017) also indicated significant interannual differences in community structure. However, significant differences during the post-DWH period were more common for comparisons separated by one or more years.

**Table 1:** Permutational multivariate analysis of variance results based on Bray-Curtis dissimilarity.

Source	df	SS	MS	Pseudo-F	P-value
Depth	1	8.15 × 10^4^	8.15 × 10^4^	41.69	<0.01*
Longitude	1	3.13 × 10^4^	3.13 × 10^4^	16.09	<0.01*
Year	8	4.28 × 10^4^	5.35 × 10^4^	1.76	<0.01*
Month/Year	26	5.80 × 10^4^	2.23 × 10^3^	1.20	0.02*
Site/Month/Year	206	3.77 × 10^5^	1.83 × 10^3^	1.20	0.17*
Residuals	7	1.07 × 10^4^	1.53 × 10^3^		
Total	249	6.01 × 10^5^			

Significant differences (α = 0.05) denoted with an asterisk (*).

**Table 2:** Post-hoc pairwise comparisons of community structure among years based on Bray-Curtis dissimilarly.

	Pre-DWH	2010	2011	2012	2013	2014	2015	2016	2017
Pre-DWH	63.0	—	—	—	—	—	—	—	—
2010	65.8*	61.6	—	—	—	—	—	—	—
2011	66.6*	65.8	67.5	—	—	—	—	—	—
2012	68.1	67.9	69.2	70.5	—	—	—	—	—
2013	67.1*	67.9*	68.7*	69.5	67.0	—	—	—	—
2014	69.5*	69.5*	70.0	70.9	68.8	71.2	—	—	—
2015	65.9*	69.1*	70.1*	70.3*	65.8*	68.5	60.8	—	—
2016	67.6*	65.8*	69.1*	69.3*	66.6*	67.9	63.3	62.0	—
2017	66.0*	65.3*	68.2*	68.1	65.3*	67.1	61.9*	60.5	60.7

Values along and below the diagonal represent within and between year dissimilarities, respectively. Significant differences (α = 0.05) denoted with an asterisk (*).

**Figure 2:**
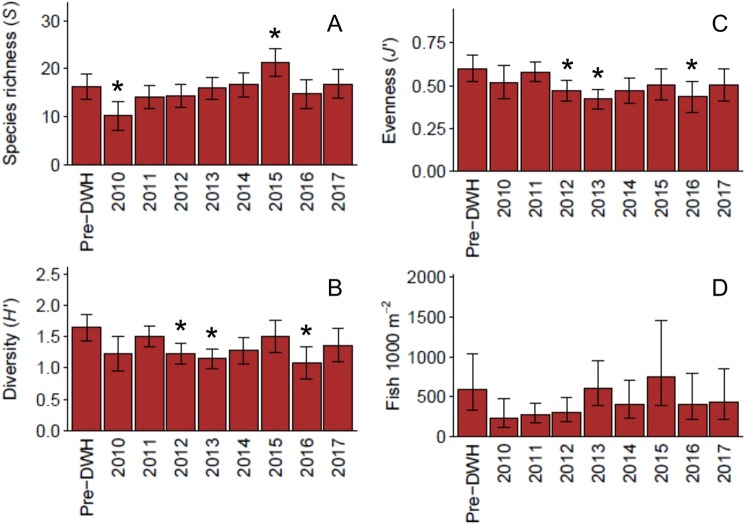
Standardized community indices or total fish density (±95% CIs) estimated from generalized linear models with corresponding 95% CIs generated through Monte Carlo simulations. An asterisk (*) denotes a significant pairwise difference between pre- and post-DWH time points (Supplemental Table S2).

All three community indices (*S*, *H*′, and *J*′), as well as total fish density, declined following the DWH (Fig. 2) with a significant decline observed for *S* (Table S2). Species richness showed an upward trend in 2011–2015, going from 14.1 to 21.3 species per site and remained comparable to our pre-DWH baseline of 16.4 in 2016 and 2017. However, *H*′ and *J*′ continued to decline and were significantly lower than pre-DWH estimates in 2012, 2013, and 2016 (Table S2). The 2010 decline in total fish density, though not statistically significant, was substantial and represented a decline of ~62%. From 2011 onward, fish density showed a slight positive trend.

### Trophic guild and species-specific trends

**Figure 3:**
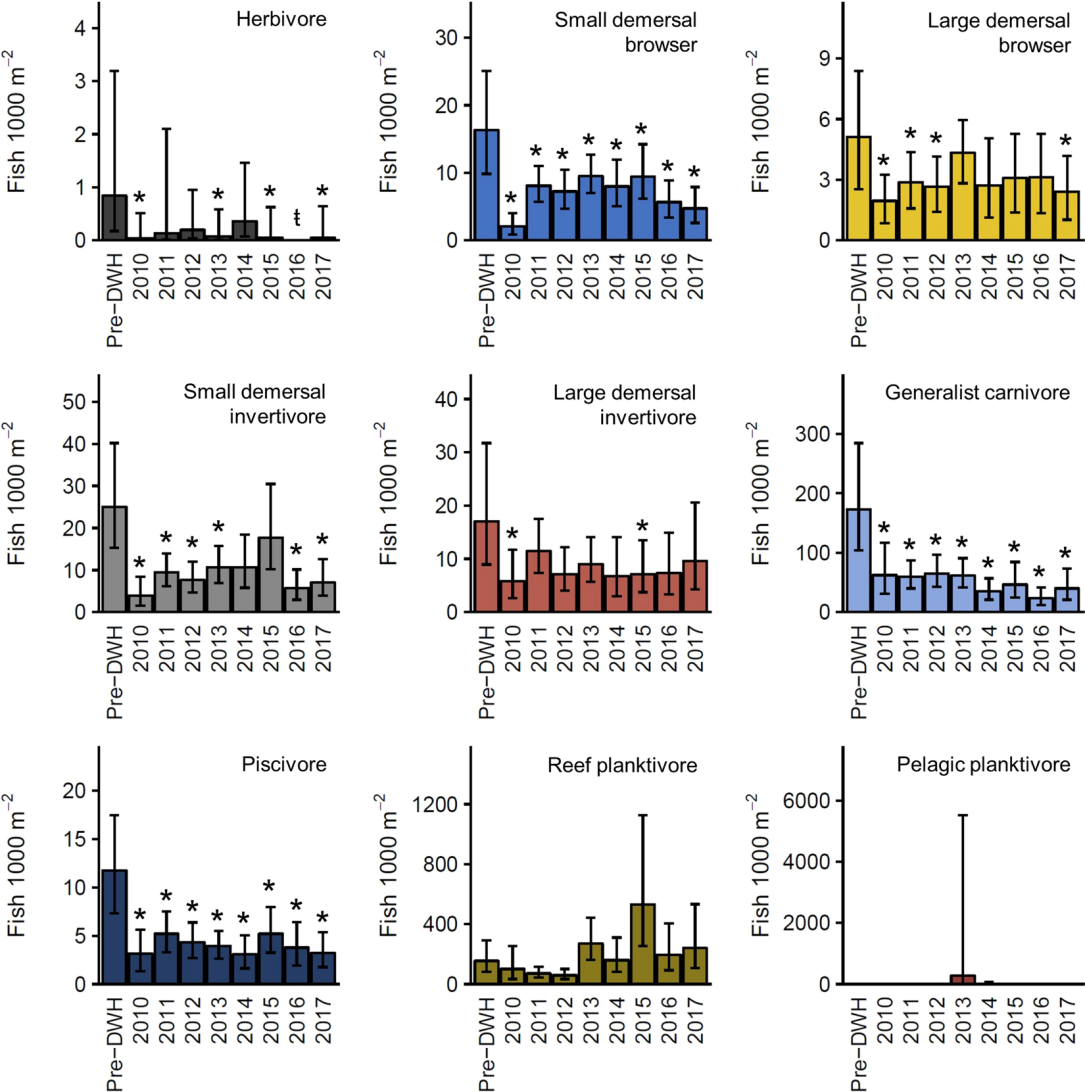
Standardized trophic guild densities (±95% CIs) estimated from generalized linear mixed effects models with corresponding 95% CIs generated through Monte Carlo simulations. A unique color was assigned to each trophic guild and this color scheme is used in subsequent figures. A stroked t (ŧ) indicates a significant pairwise difference in presence/absence between pre-DWH and post-DWH time bins (Supplemental Table S3) or complete absence. An asterisk (*) denotes a significant difference in density when present (Supplemental Table S4). Trophic guild names are provided in the top right corner of each panel.

Following the DWH, densities of all eight trophic guilds observed prior to the oil spill declined (Fig. 3). The magnitude of these declines ranged from 35 to 96% and four of the eight trophic guilds reached their lowest densities in 2010. Although we did not observed significant changes in guild presence/absence (Supplemental Table S3), the initial declines in herbivore, small and large demersal browser, small and large demersal invertivore, generalist carnivore, and piscivore densities were associated with significantly lower abundances when present (Supplemental Table S4). The 52 species for which species-specific trends were evaluated reflect this general pattern (Supplemental Table S1). Forty-six species were observed prior to the DWH and 43 declined between our pre-DWH baseline and 2010. For 29 species, these initial declines reflected either a complete absence, significant change in presence/absence, (Supplemental Table S5), or significantly lower densities when present (Supplemental Table S6).

**Figure 4:**
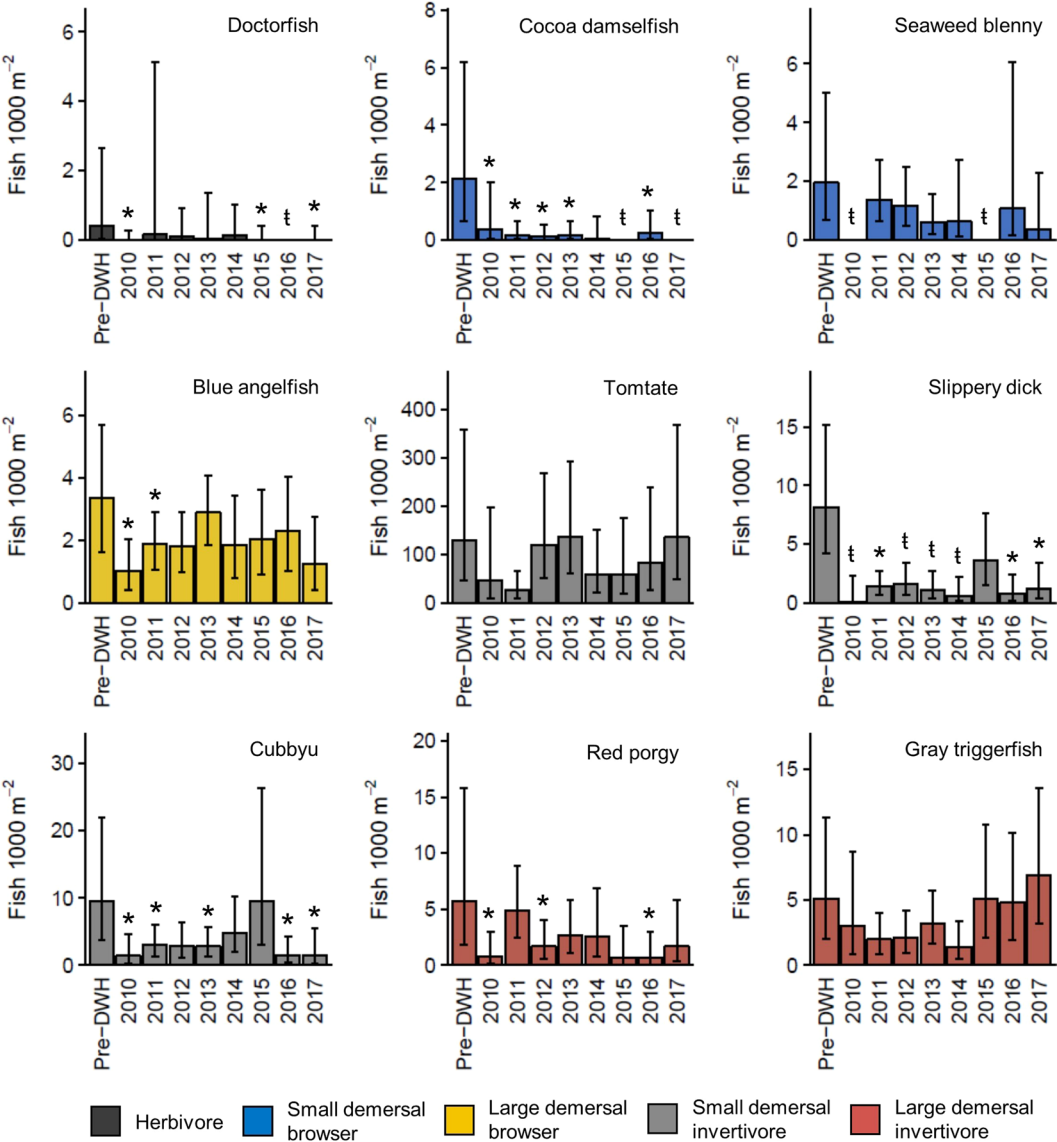
Standardized densities of representative species from the herbivore, small demersal browser, large demersal browser, small demersal invertivore, and large demersal invertivore, trophic guilds. Estimates were derived from generalized linear mixed effects models using the delta approach. Corresponding 95% CIs were generated through Monte Carlo simulations. A stroked t (ŧ) indicates a significant pairwise difference in presence/absence between pre-DWH and post-DWH time bins (Supplemental Table S5) or complete absence. An asterisk (*) denotes a significant difference in density when present (Supplemental Table S6). Species names are provided in upper right corner of each panel.

Guilds comprised of small-bodied species that forage on benthic prey showed the largest declines immediately following the DWH. Densities of herbivores, small demersal browsers, and small demersal invertivores declined by 96%, 87%, and 82%, and remained persistently low through much of the time series (Fig. 3). The decline in herbivore density almost entirely reflected doctorfish (*Acanthurus chirurgus*) abundance, while the decline in small demersal browsers resulted from lower densities of the cocoa damselfish (*Stegastes variabilis*) and seaweed blenny (*Parablennius marmoratus*) (Fig. 4). The trend displayed by small demersal invertivores was driven by species like the slippery dick (*Halichoeres bivittatus*) and cubbyu (*Paraques umbrosus*) and differed from that of the tomtate, which has a looser association with the reef structure (Fig. 4).

Declines among large-bodied species reliant on benthic production were also evident several years following the DWH, although these declines were less severe (Fig. 3). The blue angelfish (*Holacanthus bermudensis*) was the most abundant large demersal browser and drove guild-level trends (Fig. 4). The density of large demersal invertivores reflected red porgy (*Pagrus pagrus*) and gray triggerfish (*Balistes capriscus*) abundances. However, each species clearly displayed disparate patterns. The initial decline in red porgy was followed by an increase in 2011 and subsequent decline (Fig. 4). Conversely, gray triggerfish density was more variable and temporal changes were not associated with a significant difference in presence/absence or density when present.

**Figure 5:**
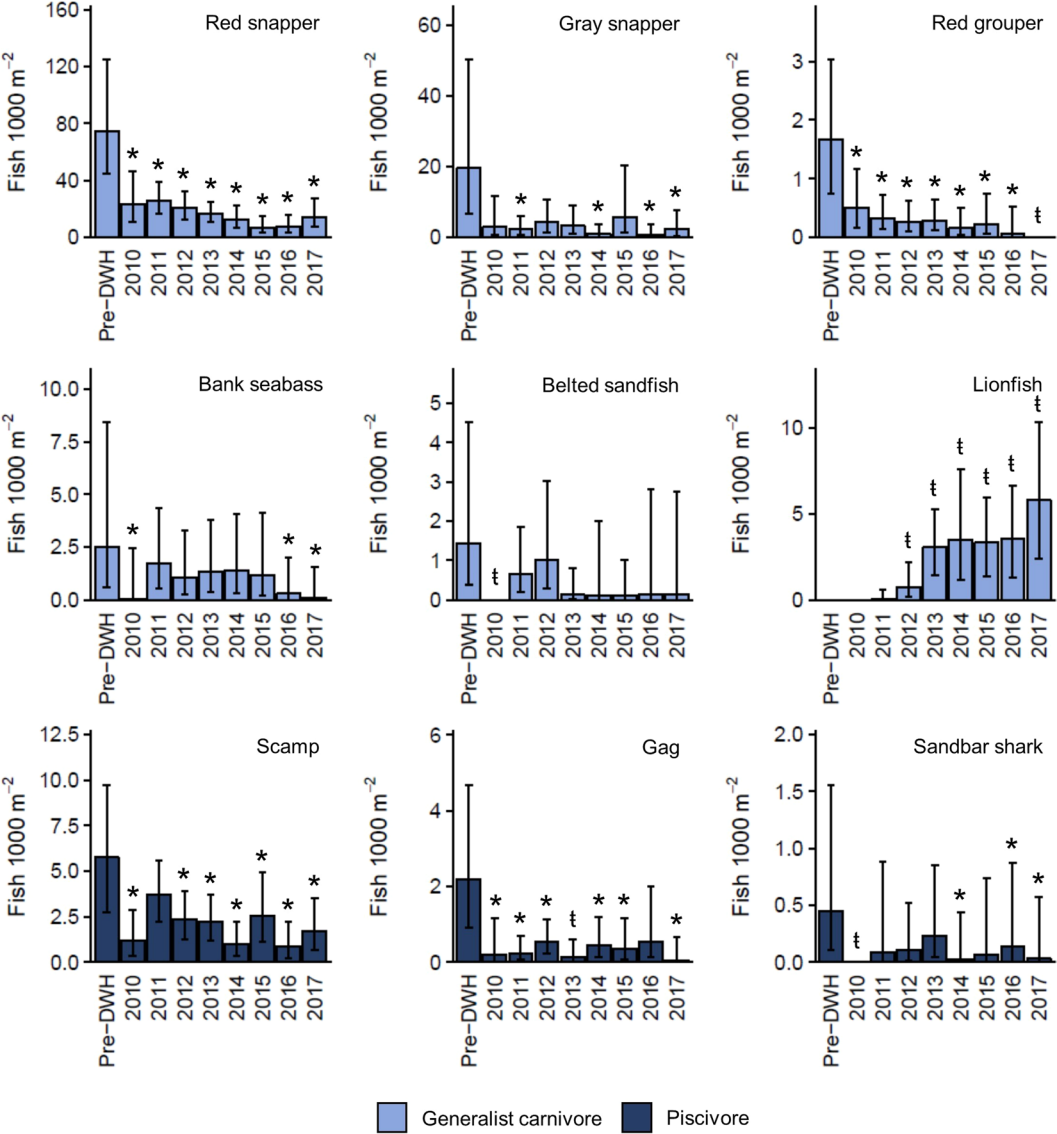
Standardized densities of representative species from the generalist carnivore and piscivore trophic guilds. Density estimates were derived from generalized linear mixed effects models using the delta approach. Corresponding 95% CIs were generated through Monte Carlo simulations. A stroked t (ŧ) indicates a significant pairwise difference in presence/absence (Supplemental Table S5) or complete absence. An asterisk (*) denotes a significant difference in density when present (Supplemental Table S6). Species names are provided in upper right corner of each panel.

Guilds representing higher trophic level consumers showed trends more similar to the small-bodied demersal guilds. Densities of generalist carnivores and piscivores declined by 64% and 73% in 2010 and densities remained persistently low thereafter (Fig. 3). For both guilds, these trends were driven by large-bodied, fisheries species (Fig. 5). Red snapper, gray snapper (*L*. *griseus*), and red grouper (*Epinephelus morio*) declined by 69%, 85%, and 70% following the DWH, and low densities persisted through 2017. The three most abundant piscivores, scamp (*Mycteroperca phenax*), gag (*M. microlepis*), and sandbar shark (*Carcharhinus plumbeus*), all declined after the spill and both scamp and gag remained at densities below pre-DWH baseline values. Densities of smaller bodied generalist carnivores, [e.g., bank seabass (*Centropristis ocyurus*) and belted sandfish (*Serranus subligarius*) (Fig. 5)] either displayed no change or failed to recover following the DWH. The one exception was the invasive lionfish (*Pterois* spp.) which was first observed in 2011 and rapidly increased through 2017 (Fig. 5).

**Figure 6:**
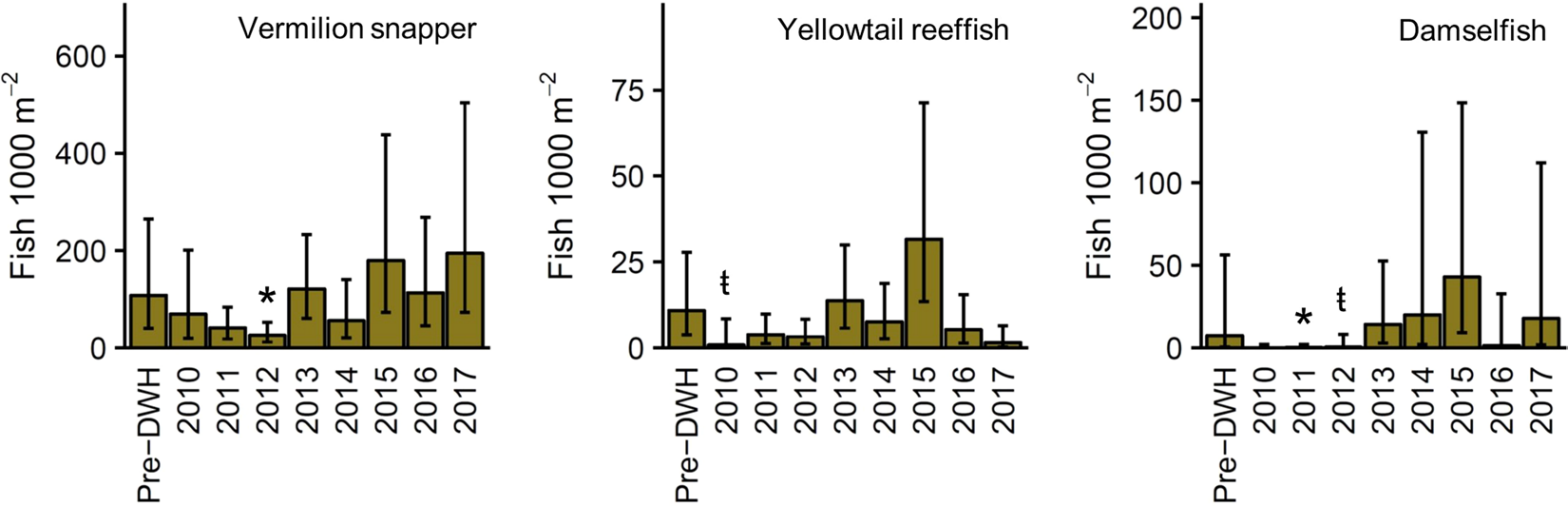
Standardized densities of reef planktivores estimated from generalized linear mixed effects models using the delta approach. Corresponding 95% CIs were generated through Monte Carlo simulations. A stroked t (ŧ) indicates a significant pairwise difference in presence/absence between pre-DWH and post-DWH time bins (Table S5) or complete absence. An asterisk (*) denotes a significant difference density when present (Table S6). Species names are provided in upper right corner of each panel.

Unlike guilds that rely on benthic forage, reef planktivore densities remained unchanged following the DWH (Fig. 3). Similarly, the downward trend through 2012 and subsequent increase were not associated with a significant difference in presence/absence or density when present (Supplemental Tables S3 and S4). The vermilion snapper was the most abundant reef planktivore and displayed a similar temporal pattern (Fig. 6). Of the nine other reef planktivores, four displayed a significant decline in presence/absence or density when present (Supplemental Table S3). However, even species that experienced declines of >90% in 2010 generally increased to pre-DWH densities [e.g., yellowtail reeffish (*Chromis enchrysura*) and damselfish (Fig. 6)]. Pelagic planktivores were not observed during our pre-DWH surveys and infrequently observed thereafter. No pelagic planktivores met our selection criteria for species-specific analysis (see Methods).

## Discussion

Our results indicate reef fish communities exhibited clear signs of negative impacts following the DWH with significant shifts in community structure and declines in species richness, diversity, evenness, and total fish density. This change in community structure was unique in that it that concomitant declines were observed for all eight trophic guilds. At no other point were similar, synchronous declines present nor were significant pairwise differences in community structure evident between successive years. The species composition of the more abundant fishes was similar before and after the spill suggesting declines in species richness resulted from an absence of rare species and changes in community structure, species diversity, evenness, and total fish density resulted from shifts in relative abundances. Declines in species richness did not persist and has remained similar to pre-DWH richness. However, lower estimates of diversity and evenness were evident several years post-spill reflecting lower densities of small demersal browsers, small demersal invertivores, generalist carnivores, and piscivores. These effects are similar to those observed among deep-sea benthic communities where declines in megafauna^83^, macrofauna^84^, meiofauna^85^, and foraminifera^86^ abundance and diversity followed the DWH. Although the most severe impacts to deep-sea benthic communities were in immediate vicinity of the wellhead^84^, the negative responses observed among decapod crustacean communities on deep bank formations^84^ and fish communities on artificial reef^70^ 100 s of km away suggests acute exposure at distant sites was of sufficient intensity to produce observable shifts.

We report declines across a range of reef fish taxa regardless of vagility, trophic position, or diet, which seem improbable without DWH-induced mortality. However, a primary challenge is identifying the extent to which declines resulted from mortality or emigration. Small fishes that live in close proximity to the reef matrix (e.g., small demersal browsers and invertivores) experienced the largest initial declines, which likely reflects a high incident of acute mortality. These species can have limited (<10 m^2^) home ranges^87^, are heavily reliant on local resource pools, and would incur the highest cost associated with emigration. These traits not only increase the probability of exposure-related mortality but also mortality associated with resource limitation^66^. Impacts on pelagic production^88,89^, increased trophic position^68,69^, and greater reliance on benthic resources^63^ suggest small demersal reef fishes experienced increased resource competition and higher predation immediately following the DWH. The effects of resource limitation were possibly exacerbated by sub-lethal exposure^37^ which can result in physiological stress leading to impaired predator avoidance and foraging ability^32,33,90^. This inference is further supported by the fact that initial declines among large-bodied reef fishes (e.g., large demersal invertivores, some generalist carnivores, and piscivores) were less severe. These species are quite mobile, exhibit varying degrees of site fidelity on natural reefs^91^, and their movements can be affected by large-scale disturbances^62,92^. Additionally, a large area (maximum = 290,000 km^2^) of the continental shelf was closed to harvest for a few months during the spill, perhaps reducing fishing-related mortality, albeit temporarily. Emigration following the DWH also may have occurred if the cost of exposure or resource limitation outweighed the benefits of staying^93,94^. For some individuals, exposure may have been sufficient to elicit movement from affected areas. For others, the response was perhaps a shift in foraging behavior^63,68^, followed by emigration as resources became more scarce. Individuals present after the DWH probably consisted of few residents that survived acute exposure and resource limitation along with new immigrants seeking more favorable conditions^93^. The fact that guilds comprised of mobile reef species (i.e., generalist carnivores and piscivores) showed little indication of recovery suggests a large number of individuals were either permanently displaced, perished (either from starvation or exposure^66^), or basal resource pools remained insufficient to support pre-DWH densities.

The effects of natural disturbances within the region (e.g., hurricanes and hypoxia) are often insufficient to produce an observable change in large, mobile reef fish abundance^95,96^. Thus, it appears the acute impact of the DWH was more severe compared to large-scale natural disturbances typical of our study area. Initial community-wide declines are, however, notably similar to the effects of harmful algal blooms that seasonally occur along the West Florida Shelf (WFS)^97–99^. The 1971 red tide event is the most well-documented case of the impacts on reef fish communities and subsequent recovery. Exposure resulted in the near extirpation of reef fishes across a >1,500 km^2^ stretch of the WFS^97^. Conspicuous^100^ and indiscriminate reef fish mortality produced clear declines in species richness, but the most pronounced effects were changes in species relative abundance^97^. Recovery followed a predictable pattern of succession initiated by the arrival of small demersal species that recruit directly to reefs, followed by a peak in abundance among early pioneers, and subsequent decline in abundance superior competitors arrived; increases among large mobile fishes that do not recruit directly to reefs occurred later^97^. Full recovery took several years, but the community that developed was nearly identical to that observed prior to the 1971 red tide. The same general pattern of community level impacts, succession, and recovery were also observed on WFS artificial reefs following a 2005 red tide event^98^, corroborating observations by Smith^97^ and others that documented successional patterns among GoM and Caribbean reef fish communities^101,102^. Although the rate of succession can vary among sites^103,104^, after seven years of post-DWH monitoring no such pattern emerged despite similarities between community members in the aforementioned studies.

A clear difference between the DWH and natural disturbances was the potential for chronic PAH exposure, which can have long-term, higher-order impacts even at sub-lethal levels^105^. While sedimentation of contaminated marine snow was the primary vector transporting oil and dispersants to the benthos, this phenomenon was patchy and mostly concentrated off the continental shelf and west of our sampling area^106,107^. As a result, sediment concentrations of total petroleum and polycyclic aromatic hydrocarbons (PAHs) were typical of background levels within our study area in August 2010^54^. Nonetheless, examination of reef fish tissue samples indicated elevated levels of PAH concentrations in liver and PAH metabolites in bile persisted for years following the DWH^37,65,108^, thus providing evidence of continual reef fish exposure to toxic petroleum compounds for some time after the spill despite uncertainties about the mechanism of exposure.

The fact that reef fish density remained low for a number of years following the DWH suggests indirect food web effects have played an important role as well^66^. Consumption of swarming zooplankton by red snapper declined markedly following the DWH, a possible reflection of an initial reduction in pelagic production^66^. The reduction in zooplankton was balanced by increased foraging on demersal fishes and invertebrates^63^. This increased reliance on benthic resources continued over weeks and months following DWH, as indicated by declines in δ^34^S^63,68^. The associated enrichment of δ^15^N persisted for several years following the DWH^63,68^, and similar long-term shifts in δ^15^N were observed for gray triggerfish, tomtate, red porgy, and vermilion snapper concurrent with declines in δ^13^C, indicative of petrocarbon cycling through the food web^68,109^. The 2010 shifts in trophic position and community-wide declines that extended into 2011 also pre-date the arrival and rapid expansion of the invasive lionfish and are in general agreement with previous reports that community-wide impacts following the DWH occurred prior to the nGoM lionfish invasion^70^.

Although the role of food web effects resulting from a shift in resource availability is apparent, the success of the invasive lionfish may also be an important factor suppressing community recovery. Numerous studies have documented community-level effects of lionfish^110,111^ via predation^112,113^ and competition^114,115^. Impacts are typically most evident among small demersal reef fishes that recruit directly to reef habitats^70^ and vulnerable to predation both as adults^112,116^ and newly-settled recruits^113^. The stunted recovery of small demersal invertivores and browsers, despite their capacity for rapid recolonization following mass mortality events^97^ and the continued declines among native predators, provides a clear indication that the success of the invasive lionfish is affecting the response among native, small demersal fishes. How lionfish may be affecting the recovery of fisheries species has not been evaluated, but competition between lionfish and native predators (e.g., groupers, snappers, and jacks) may affect population productivity^117^. Taxonomic resolution of dietary data for fisheries species is often poor and presents challenges when attempting to capture competitive interactions in systems with diverse species assemblages^117^. Diet information for the majority of small demersal reef fish is also lacking, and the potential food web effects emanating from low densities of small demersal species remains unknown.

The changes in community structure, particularly the persistently low densities among certain groups, provides a clear indication of lasting, community-wide impacts. The available evidence suggests initial declines in 2010 likely reflected both mortality and emigration resulting from exposure and resource limitation. Mortality due to direct or indirect effects of the spill likely drove initial declines of small demersal species, while large-bodied consumers were more likely to be permanently displaced or to suffer delayed mortality. Community-wide declines into 2011 were indicative of protracted resource limitation. However, the lack of recovery in small demersal reef fishes from 2012 onward may, to some extent, reflect top-down pressure from lionfish. How community shifts have altered the flow of energy to higher trophic level fisheries species or impacted system resilience remains uncertain.

A clear challenge moving forward is identifying the underlying mechanisms driving these patterns, estimating the relative impacts of individual stressors (e.g., exposure, resource limitation, lionfish invasion, fisheries harvest, and food web effects), and developing management strategies to facilitate recovery. Efforts along these lines appear particularly relevant considering the dramatic declines observed for higher trophic position consumers that not only serve important ecological roles^118^ but also provide numerous economic^119^ and cultural^120^ benefits. Many of the ecosystems services we ascribe to reef fish assemblages in the nGoM are inextricably linked to fisheries harvest^119,120^, and fishery-dependent data and assessment of fishery stocks should enable tracking resilience among those species. However, continued funding for fishery-independent surveys, such as the ROV work that forms that basis of the analyses presented herein, is critical to assess the long-term effects of the DWH, lionfish invasion, and the potential for resiliency in the nGoM ecosystem.

## Supplementary information

Supplemental Materials.

Appendix A.

## Data Availability

Data are available through the Gulf of Mexico Research Initiative Information & Data Cooperative (GRIIDC) at https://data.gulfresearchinitiative.org under DOI 10.7266/N72J68SF and 10.7266/n7-n4j3-0a26. The remaining portion of the time series can be obtained from the corresponding author upon request.
