# Author Correction: Changes in Reef Fish Community Structure Following the Deepwater Horizon Oil Spill

**DOI:** 10.1038/s41598-020-68557-3

**Published:** 2020-07-07

**Authors:** Justin P. Lewis, Joseph H. Tarnecki, Steven B. Garner, David D. Chagaris, William F. Patterson

**Affiliations:** 1 University of Florida, Fisheries and Aquatic Sciences, Gainesville, Florida 32608 USA; 2 University of Florida, Nature Coast Biological Station, Cedar Key, FL 32625 USA

Correction to: *Scientific Reports*, 10.1038/s41598-020-62574-y published online 09 April 2020.

In this Article, the Data availability section is incomplete. The correct version of this statement can be seen below:

“Data are available through the Gulf of Mexico Research Initiative Information & Data Cooperative (GRIIDC) at https://data.gulfresearchinitiative.org under DOI 10.7266/N72J68SF, 10.7266/Y9N4TPS3, and 10.7266/n7-n4j3-0a26. The remaining portion of the time series can be obtained from the corresponding author upon request.”

