# Supplementary material for: Changes in Reef Fish Community Structure Following the Deepwater Horizon Oil Spill
Source: Sci Rep. 2020 Apr 9;10:5621. doi: 10.1038/s41598-020-62574-y (PMC7145834; doi:10.1038/s41598-020-62574-y)

*The following supplement accompanies the article*

**Changes in Reef Fish Community Structure Following the Deepwater Horizon Oil Spill**

**Justin P. Lewis^*^, Joseph H. Tarnecki, Steven B. Garner, David D. Chagaris, and William F. Patterson III**

^*^corresponding author: [justin.lewis@ufl.edu](mailto:justin.lewis@ufl.edu)

**Table S1.** Species-specific standardized pre-*Deepwater Horizon* densities (Pre-DWH) and percent change (%) following DWH. Standardized densities were estimated from generalized linear mixed effects models (GLMM) for 52 fish species. Pre-DWH densities were back transformed into original measurement units (Fish × 1000m^-2^). Cell color represents either a decrease (red), no change (white), or increase (green) in fish density. Shading is along a continuum from -100 to 100%. A stroked t (ŧ) denotes a significant (α=0.05) pairwise difference in the binomial GLMM (see Table S5). An asterisk is used to identify significant pairwise differences in the log-normal GLMM (see Table S6).

| **Trophic Guild** | **Scientific Name** | **Pre-DWH** | **2010** | **2011** | **2012** | **2013** | **2014** | **2015** | **2016** | **2017** |
| --- | --- | --- | --- | --- | --- | --- | --- | --- | --- | --- |
| Herbivore | *Acanthurus chirurgus* | 0.39 | -99.9* | -61 | -73.8 | -88.2 | -66.4 | -98.7* | -100.0ŧ | -98.6* |
| Small Demersal Browser | *Canthigaster rostrata* | 0.76 | -100.0ŧ | -76.6* | -67.9* | 22.9* | -13.6* | 292.1ŧ | -63.2* | -56.9* |
|  | *Chaetodon ocellatus* | 1.31 | -82.6 | -57.9 | -38.5 | -63.1 | -70.1 | -53.4 | -86.6 | -15.2 |
|  | *Chaetodon sedentarius* | 1.85 | -75.8* | 3.5 | -30.3* | 36 | -24.2* | 7.5* | -39.5* | -93.1* |
|  | *Parablennius marmoreus* | 1.95 | -100.0ŧ | -29.2 | -40.7 | -69.9 | -68 | -100.0ŧ | -44.8 | -82.3 |
|  | *Prognathodes aya* | 0.52 | -97.5* | -41.8* | -55.5* | -40.3* | -41.5 | 2.5* | -100.0ŧ | -67.8* |
|  | *Stegastes leucostictus* | 1.18 | -96.7 | -78.2 | -75.5 | -3.8 | 21.7 | -10.7* | -53.7 | -64.6 |
|  | *Stegastes variabilis* | 2.15 | -83.0* | -93.2* | -94.2* | -92.4* | -97.5 | -100.0ŧ | -88.7* | -100.0ŧ |
| Large Demersal Browser | *Aluterus monoceros* | 0.00 | 0.0 | 1 | 4.2 | 7.1 | 9.1 | 2.1 | 0 | 2.2 |
|  | *Holacanthus bermudensis* | 3.34 | -69.2* | -44.1* | -45.7 | -13.4 | -44.5 | -39.4 | -31.1 | -62.8 |
|  | *Holacanthus ciliaris* | 0.45 | -51.5 | -50.4* | -90.5* | -72.6* | -83.1* | -92.8* | -82.2 | -90.3* |
| Small Demersal Invertivore | *Bodianus pulchellus* | 0.86 | -37.8* | -60.7 | -59.6 | -8.2* | -73.3* | -22.1* | -66.8* | -74.2* |
|  | *Bodianus rufus* | 0.13 | -100.0ŧ | -82.2 | -85.8* | -41.5 | -63.6* | -68.3* | 57.8* | -93.7* |
|  | *Equetus lanceolatus* | 0.57 | -89.1* | -82.3 | -88.5 | -9.6 | -52.5* | -37.2 | -45.4 | 31* |
|  | *Haemulon aurolineatum* | 129.53 | -64.3 | -79.3 | -8.4 | 5 | -55.8 | -54.9 | -36.3 | 4 |
|  | *Halichoeres bivittatus* | 8.15 | -98.6ŧ | -83.0* | -80.2ŧ | -86.4ŧ | -92.5ŧ | -56.0 | -90.2* | -85.0* |
|  | *Holocentrus adscensionis* | 0.28 | -100.0ŧ | -40.8 | -6.3 | 16.3 | -88.2 | 65.4 | -83.2 | -71.9 |
|  | *Holocentrus rufus* | 0.00 | 0.0 | 6.9 | 7.1 | 0 | 59.2 | 0 | 17.1 | 10.9 |
|  | *Pareques acuminatus* | 0.80 | -100.0ŧ | -47.4* | -89.2* | 22.2* | -89.2* | -100.0ŧ | -81.0 | -100.0ŧ |
|  | *Pareques umbrosus* | 9.50 | -85.5* | -68.4* | -69.9 | -70.3* | -50.3 | 0.6 | -85.2* | -84.3* |
| Large Demersal Invertivore | *Archosargus probatocephalus* | 0.00 | -100.0ŧ | -35.0 | 340.9 | -64.4 | 375.7 | -85.5 | -100.0ŧ | -86.4 |
|  | *Balistes capriscus* | 5.11 | -40.4 | -61.1 | -58.8 | -37.2 | -72.4 | 0.2 | -6.1 | 35.8 |
|  | *Chilomycterus schoepfi* | 0.00 | 0 | 9.5 | 12.9 | 0 | 18.2 | 12.9 | 24.1 | 5.9 |
|  | *Pagrus pagrus* | 5.76 | -86.1* | -14.8 | -70.9* | -53.9 | -55.2 | -88.0 | -88.2* | -70.7 |

**Table S1. Continued.**

| **Trophic Guild** | **Scientific Name** | **Pre-DWH** | **2010** | **2011** | **2012** | **2013** | **2014** | **2015** | **2016** | **2017** |
| --- | --- | --- | --- | --- | --- | --- | --- | --- | --- | --- |
| Generalist Carnivore | *Caranx crysos* | 8.52 | -84.0 | -99.3 | -46.3 | -75.4 | 263.8 | -87.2 | -100.0ŧ | -94.9 |
|  | *Centropristis ocyurus* | 2.52 | -97.8 | -31.5 | -57.9 | -47.1 | -44.9 | -53.7 | -88.5* | -96.4* |
|  | *Diplectrum formosum* | 0.00 | 0.0 | 9.0 | 28.8 | 30.8 | 7.2 | 90.2ŧ | 0.0 | 0.0 |
|  | *Epinephelus cruentatus* | 0.12 | -100.0ŧ | -84.6 | -54.7 | -52.9 | -44.1 | 64.2 | -73.0 | 68.0 |
|  | *Epinephelus morio* | 1.66 | -69.9* | -80.1* | -84.2* | -82.6* | -90.7* | -86.5* | -95.8* | -100.0ŧ |
|  | *Liopropoma eukrines* | 0.09 | 15.1* | 141.2 | 300.2 | 220.9 | 420.9 | 985.3ŧ | -54.2* | 127.5 |
|  | *Lutjanus campechanus* | 74.59 | -68.9* | -65.5* | -72.4* | -78.1* | -83.1* | -90.7* | -89.6* | -81.1* |
|  | *Lutjanus griseus* | 19.49 | -84.9 | -87.5* | -78.2 | -83.5 | -95.1* | -70.8 | -96.4* | -88.5* |
|  | *Lutjanus synagris* | 4.21 | -26.1 | -96.6ŧ | -84.2 | -49.1 | -94.5 | -83.6 | -92.8 | -80.3 |
|  | *Pterois volitans* | 0.00 | 0.0 | 1.7 | 76.1ŧ | 305.1ŧ | 349.6ŧ | 332.4ŧ | 353.7ŧ | 580.2ŧ |
|  | *Rypticus maculatus* | 1.06 | -80.0 | 28.2 | -24.3 | -14.3 | -10.9 | 19.9 | -81.5 | -28.9 |
|  | *Seriola dumerili* | 13.30 | 53.8 | 21.5 | -35.6 | -65.6 | -86.5 | -59.6 | -90.1 | -77.6 |
|  | *Seriola rivoliana* | 3.60 | -100.0ŧ | -91.6ŧ | -88.7* | -81.2 | -94.0 | -47.9 | -74.1 | -92.9* |
|  | *Serranus phoebe* | 1.50 | -79.4* | -73.6 | 2.7 | -70.1* | -27.3* | -5.9 | -73.4* | -58.9* |
|  | *Serranus subligarius* | 1.44 | -100.0ŧ | -54.9 | -30.6 | -90.5 | -91.7 | -92.1 | -89.5 | -89.4 |
| Piscivore | *Carcharhinus plumbeus* | 0.45 | -100.0ŧ | -81.1 | -75.5 | -48.0 | -94.2* | -84.9 | -69.1* | -92.7* |
|  | *Mycteroperca microlepis* | 2.18 | -90.3* | -89.3* | -75.5* | -93.9ŧ | -79.0* | -84.0* | -74.6 | -97.9* |
|  | *Mycteroperca phenax* | 5.75 | -79.3* | -35.7 | -59.3* | -61.2* | -82.8* | -55.3* | -85.2* | -70.4* |
| Reef Planktivore | *Apogon pseudomaculatus* | 0.74 | -79.0 | 34.9 | -84.3* | 14.1 | -52.9 | -16.7 | -14.5 | -100.0ŧ |
|  | *Baldwinella aureorubens* | 0.00 | 0.0 | 0.4 | 0.0 | 3.6 | 2.0 | 1260.9* | 3.4 | 5.4 |
|  | *Chromis enchrysura* | 10.86 | -92.3ŧ | -64.7 | -70.3 | 26.3 | -30.2 | 190.9 | -51.3 | -85.9 |
|  | *Paranthias furcifer* | 0.44 | -90.5 | 0.8 | -48.4 | -89.8 | -70.2 | 351.5 | -100.0ŧ | -100.0ŧ |
|  | *Priacanthus arenatus* | 1.74 | -77.4* | 82.8 | 133.6 | 143.7 | -18.9 | -89.5 | -100.0ŧ | -73.6 |
|  | *Pristigenys alta* | 0.69 | -100.0ŧ | -96.2ŧ | -100.0ŧ | -91.8 | -20.6 | 536.0ŧ | 669.3ŧ | 504.8ŧ |
|  | *Pronotogrammus martinicensis* | 0.01 | 15.4 | 514.3 | 344.2 | 1686.2 | 1622.7 | 4939.5 | 134.6 | 154.5 |
|  | *Rhomboplites aurorubens* | 107.84 | -35.7 | -61.9* | -76.3 | 12.5 | -48.3 | 66.6 | 5.0 | 80.5 |
|  | *Stegastes partitus* | 0.20 | -98.6* | -100.0ŧ | -100.0ŧ | -95.6 | -78.3 | -45.1 | -97.9 | -100.0ŧ |
|  | Damselfish | 7.39 | -99.8 | -97.0* | -93.7ŧ | 91.8 | 170.5 | 480.7 | -81.0 | 141.4 |
| -- | Total Fish | 555.54 | -61.8 | -54.4 | -35.4 | 7.2 | -38.3 | 25.0 | -32.8 | -29.4 |

**Table S2.** Dunnett’s multiple comparisons of generalize linear models assessing changes in species richness, diversity, and evenness and total fish density following the *Deepwater Horizon* oil spill (DHW). Asterisks (*) denote significant differences at α=0.05.

| Community index | Comparison | Estimate | SE | z | *P* |
| --- | --- | --- | --- | --- | --- |
| Species richness | 2010 - Pre-DWH | -6.19 | 1.48 | -4.19 | <0.01* |
|  | 2011 - Pre-DWH | -2.23 | 1.13 | -1.97 | 0.25 |
|  | 2012 - Pre-DWH | -2.07 | 1.15 | -1.81 | 0.33 |
|  | 2013 - Pre-DWH | -0.45 | 1.12 | -0.4 | 1 |
|  | 2014 - Pre-DWH | 0.28 | 1.27 | 0.22 | 1 |
|  | 2015 - Pre-DWH | 4.93 | 1.44 | 3.42 | <0.01* |
|  | 2016 - Pre-DWH | -1.64 | 1.44 | -1.14 | 0.8 |
|  | 2017 - Pre-DWH | 0.42 | 1.47 | 0.29 | 1 |
| Species diversity  (Shannon-Weiner *H*’) | 2010 - Pre-DWH | -0.42 | 0.16 | -2.6 | 0.06 |
|  | 2011 - Pre-DWH | -0.14 | 0.12 | -1.15 | 0.8 |
|  | 2012 - Pre-DWH | -0.42 | 0.12 | -3.36 | <0.01* |
|  | 2013 - Pre-DWH | -0.5 | 0.12 | -4.09 | <0.01* |
|  | 2014 - Pre-DWH | -0.37 | 0.14 | -2.65 | 0.05 |
|  | 2015 - Pre-DWH | -0.13 | 0.16 | -0.84 | 0.95 |
|  | 2016 - Pre-DWH | -0.56 | 0.16 | -3.55 | <0.01* |
|  | 2017 - Pre-DWH | -0.28 | 0.16 | -1.77 | 0.36 |
| Species evenness  (Pielou’s *J*’) | 2010 - Pre-DWH | -0.08 | 0.06 | -1.37 | 0.64 |
|  | 2011 - Pre-DWH | -0.02 | 0.04 | -0.43 | 1 |
|  | 2012 - Pre-DWH | -0.13 | 0.04 | -2.94 | 0.02* |
|  | 2013 - Pre-DWH | -0.18 | 0.04 | -4.11 | <0.01* |
|  | 2014 - Pre-DWH | -0.13 | 0.05 | -2.64 | 0.05 |
|  | 2015 - Pre-DWH | -0.09 | 0.06 | -1.66 | 0.43 |
|  | 2016 - Pre-DWH | -0.16 | 0.06 | -2.94 | 0.02* |
|  | 2017 - Pre-DWH | -0.09 | 0.06 | -1.66 | 0.43 |
| Total fish density | 2010 - Pre-DWH | -0.92 | 0.39 | -2.35 | 0.11 |
|  | 2011 - Pre-DWH | -0.78 | 0.3 | -2.6 | 0.06 |
|  | 2012 - Pre-DWH | -0.65 | 0.3 | -2.14 | 0.17 |
|  | 2013 - Pre-DWH | 0.04 | 0.3 | 0.13 | 1 |
|  | 2014 - Pre-DWH | -0.36 | 0.34 | -1.06 | 0.85 |
|  | 2015 - Pre-DWH | 0.25 | 0.38 | 0.66 | 0.99 |
|  | 2016 - Pre-DWH | -0.35 | 0.38 | -0.93 | 0.92 |
|  | 2017 - Pre-DWH | -0.31 | 0.39 | -0.79 | 0.97 |

**Table S3.** Results from Dunnett’s multiple comparisons of linear mixed effects model comparing trophic guild presence/absence between pre- and post-DWH time points. No pair-wise differences were significant (α = 0.05).

| Trophic guild | Comparison | Estimate | SE | *z* | *P* |
| --- | --- | --- | --- | --- | --- |
| Herbivore | 2010 - Pre-DWH | -1.78 | 1.28 | -1.39 | 0.64 |
|  | 2011 - Pre-DWH | -3.1 | 1.24 | -2.51 | 0.07 |
|  | 2012 - Pre-DWH | -1.12 | 0.84 | -1.32 | 0.69 |
|  | 2013 - Pre-DWH | -2.37 | 1.01 | -2.35 | 0.11 |
|  | 2014 - Pre-DWH | -0.37 | 0.88 | -0.42 | 1 |
|  | 2015 - Pre-DWH | -1.9 | 1.28 | -1.48 | 0.57 |
|  | 2016 - Pre-DWH | -- | -- | -- | -- |
|  | 2017 - Pre-DWH | -1.81 | 1.29 | -1.41 | 0.63 |
| Small Demersal Browser | 2010 - Pre-DWH | -0.99 | 0.86 | -1.15 | 0.81 |
|  | 2011 - Pre-DWH | 0.45 | 0.75 | 0.6 | 0.99 |
|  | 2012 - Pre-DWH | -0.25 | 0.72 | -0.35 | 1 |
|  | 2013 - Pre-DWH | 1.08 | 0.77 | 1.4 | 0.64 |
|  | 2014 - Pre-DWH | 0.42 | 0.82 | 0.51 | 1 |
|  | 2015 - Pre-DWH | 2.65 | 1.34 | 1.97 | 0.26 |
|  | 2016 - Pre-DWH | 0.86 | 1.01 | 0.85 | 0.96 |
|  | 2017 - Pre-DWH | 0.22 | 0.95 | 0.23 | 1 |
| Large Demersal Browser | 2010 - Pre-DWH | 0.29 | 0.86 | 0.34 | 1 |
|  | 2011 - Pre-DWH | -0.15 | 0.65 | -0.23 | 1 |
|  | 2012 - Pre-DWH | -0.3 | 0.66 | -0.46 | 1 |
|  | 2013 - Pre-DWH | 0.63 | 0.66 | 0.95 | 0.92 |
|  | 2014 - Pre-DWH | -0.7 | 0.73 | -0.96 | 0.91 |
|  | 2015 - Pre-DWH | 0.11 | 0.85 | 0.13 | 1 |
|  | 2016 - Pre-DWH | 0.11 | 0.85 | 0.13 | 1 |
|  | 2017 - Pre-DWH | 0.04 | 0.87 | 0.04 | 1 |
| Small Demersal Invertivore  (with tomtate) | 2010 - Pre-DWH | -1.37 | 1.18 | -1.16 | 0.75 |
|  | 2011 - Pre-DWH | -0.61 | 1 | -0.61 | 0.99 |
|  | 2012 - Pre-DWH | -1.4 | 0.96 | -1.45 | 0.54 |
|  | 2013 - Pre-DWH | -1.05 | 0.97 | -1.09 | 0.8 |
|  | 2014 - Pre-DWH | -1.46 | 1.02 | -1.44 | 0.55 |
|  | 2015 - Pre-DWH | 0.84 | 1.48 | 0.57 | 0.99 |
|  | 2016 - Pre-DWH | -0.26 | 1.27 | -0.21 | 1 |
|  | 2017 - Pre-DWH | -0.29 | 1.28 | -0.23 | 1 |

**Table S3. Continued**

| Trophic guild | Comparison | Estimate | SE | *z* | *P* |
| --- | --- | --- | --- | --- | --- |
| Small Demersal Invertivore (without tomtate) | 2010 - Pre-DWH | -1.75 | 0.97 | -1.81 | 0.32 |
|  | 2011 - Pre-DWH | -0.73 | 0.83 | -0.87 | 0.93 |
|  | 2012 - Pre-DWH | -1.3 | 0.82 | -1.59 | 0.46 |
|  | 2013 - Pre-DWH | -0.66 | 0.83 | -0.8 | 0.95 |
|  | 2014 - Pre-DWH | -1 | 0.88 | -1.13 | 0.79 |
|  | 2015 - Pre-DWH | 1.49 | 1.39 | 1.07 | 0.82 |
|  | 2016 - Pre-DWH | -0.27 | 1.07 | -0.25 | 1 |
|  | 2017 - Pre-DWH | 0.43 | 1.17 | 0.37 | 1 |
| Large Demersal Invertivore | 2010 - Pre-DWH | 0.52 | 0.95 | 0.55 | 1 |
|  | 2011 - Pre-DWH | 0.87 | 0.69 | 1.27 | 0.74 |
|  | 2012 - Pre-DWH | -0.58 | 0.64 | -0.91 | 0.94 |
|  | 2013 - Pre-DWH | 0.38 | 0.65 | 0.58 | 1 |
|  | 2014 - Pre-DWH | -1.01 | 0.69 | -1.46 | 0.59 |
|  | 2015 - Pre-DWH | 1.96 | 1.19 | 1.65 | 0.45 |
|  | 2016 - Pre-DWH | 0.08 | 0.83 | 0.1 | 1 |
|  | 2017 - Pre-DWH | -0.03 | 0.84 | -0.04 | 1 |
| Generalist Carnivore | 2010 - Pre-DWH | -0.73 | 1.5 | -0.49 | 1 |
|  | 2011 - Pre-DWH | 0.79 | 1.49 | 0.53 | 1 |
|  | 2012 - Pre-DWH | 0.04 | 1.31 | 0.03 | 1 |
|  | 2013 - Pre-DWH | 0.1 | 1.3 | 0.08 | 1 |
|  | 2014 - Pre-DWH | 0.22 | 1.49 | 0.15 | 1 |
|  | 2015 - Pre-DWH | -0.34 | 1.51 | -0.23 | 1 |
|  | 2016 - Pre-DWH | -0.34 | 1.51 | -0.23 | 1 |
|  | 2017 - Pre-DWH | -0.38 | 1.51 | -0.25 | 1 |
| Piscivore | 2010 - Pre-DWH | -1 | 1.01 | -0.99 | 0.85 |
|  | 2011 - Pre-DWH | -0.94 | 0.78 | -1.21 | 0.69 |
|  | 2012 - Pre-DWH | -1.01 | 0.78 | -1.3 | 0.63 |
|  | 2013 - Pre-DWH | -0.62 | 0.78 | -0.8 | 0.94 |
|  | 2014 - Pre-DWH | -1.23 | 0.84 | -1.47 | 0.51 |
|  | 2015 - Pre-DWH | 0.79 | 1.09 | 0.73 | 0.96 |
|  | 2016 - Pre-DWH | -0.76 | 0.93 | -0.82 | 0.93 |
|  | 2017 - Pre-DWH | -0.37 | 0.96 | -0.38 | 1 |
| Reef Planktivore | 2010 - Pre-DWH | -2.56 | 1.62 | -1.58 | 0.44 |
|  | 2011 - Pre-DWH | -0.37 | 1.27 | -0.29 | 1 |
|  | 2012 - Pre-DWH | -1.54 | 1.27 | -1.21 | 0.71 |
|  | 2013 - Pre-DWH | -1.26 | 1.27 | -1 | 0.86 |
|  | 2014 - Pre-DWH | -0.48 | 1.33 | -0.36 | 1 |
|  | 2015 - Pre-DWH | 1.91 | 1.65 | 1.16 | 0.75 |
|  | 2016 - Pre-DWH | 1.91 | 1.65 | 1.16 | 0.75 |
|  | 2017 - Pre-DWH | 0.66 | 1.52 | 0.43 | 1 |

**Table S4.** Results from Dunnett’s multiple comparisons of the log-normal linear mixed effects model comparing pre- and post-DWH time points. Asterisks (*) denote significant differences at α = 0.05.

| Trophic guild | Comparison | Estimate | SE | *z* | *P* |
| --- | --- | --- | --- | --- | --- |
| Herbivore | 2010 - Pre-DWH | -1.53 | 0.44 | -3.47 | <0.01* |
|  | 2011 - Pre-DWH | 1.07 | 0.65 | 1.65 | 0.46 |
|  | 2012 - Pre-DWH | -0.45 | 0.35 | -1.31 | 0.7 |
|  | 2013 - Pre-DWH | -0.27 | 0.35 | -0.76 | 0.97 |
|  | 2014 - Pre-DWH | -0.56 | 0.38 | -1.48 | 0.58 |
|  | 2015 - Pre-DWH | -1.2 | 0.44 | -2.71 | 0.04* |
|  | 2016 - Pre-DWH | -- | -- | -- | -- |
|  | 2017 - Pre-DWH | -1.2 | 0.44 | -2.71 | 0.04* |
| Small Demersal Browser | 2010 - Pre-DWH | -1.78 | 0.29 | -6.13 | <0.01* |
|  | 2011 - Pre-DWH | -0.79 | 0.21 | -3.79 | <0.01* |
|  | 2012 - Pre-DWH | -0.77 | 0.22 | -3.56 | <0.01* |
|  | 2013 - Pre-DWH | -0.69 | 0.2 | -3.43 | <0.01* |
|  | 2014 - Pre-DWH | -0.79 | 0.24 | -3.35 | <0.01* |
|  | 2015 - Pre-DWH | -0.76 | 0.25 | -3.05 | 0.02* |
|  | 2016 - Pre-DWH | -1.17 | 0.26 | -4.53 | <0.01* |
|  | 2017 - Pre-DWH | -1.28 | 0.27 | -4.69 | <0.01* |
| Large Demersal Browser | 2010 - Pre-DWH | -1.06 | 0.21 | -5.04 | <0.01* |
|  | 2011 - Pre-DWH | -0.53 | 0.18 | -3.02 | 0.02* |
|  | 2012 - Pre-DWH | -0.54 | 0.18 | -3.02 | 0.02* |
|  | 2013 - Pre-DWH | -0.37 | 0.17 | -2.18 | 0.15 |
|  | 2014 - Pre-DWH | -0.32 | 0.21 | -1.52 | 0.51 |
|  | 2015 - Pre-DWH | -0.55 | 0.22 | -2.46 | 0.08 |
|  | 2016 - Pre-DWH | -0.53 | 0.22 | -2.42 | 0.09 |
|  | 2017 - Pre-DWH | -0.77 | 0.23 | -3.42 | <0.01* |
| Small Demersal Invertivore  (with tomtate) | 2010 - Pre-DWH | -1.38 | 0.59 | -2.33 | 0.12 |
|  | 2011 - Pre-DWH | -1.41 | 0.45 | -3.13 | 0.01* |
|  | 2012 - Pre-DWH | -0.29 | 0.46 | -0.62 | 0.99 |
|  | 2013 - Pre-DWH | -0.12 | 0.45 | -0.28 | 1 |
|  | 2014 - Pre-DWH | -0.37 | 0.53 | -0.7 | 0.98 |
|  | 2015 - Pre-DWH | -0.56 | 0.56 | -1.01 | 0.89 |
|  | 2016 - Pre-DWH | -0.6 | 0.57 | -1.05 | 0.86 |
|  | 2017 - Pre-DWH | -0.03 | 0.58 | -0.05 | 1 |

**Table S4. Continued**

| Trophic guild | Comparison | Estimate | SE | *z* | *P* |
| --- | --- | --- | --- | --- | --- |
| Small Demersal Invertivore  (without tomtate) | 2010 - Pre-DWH | -1.5 | 0.37 | -4.06 | <0.01* |
|  | 2011 - Pre-DWH | -0.89 | 0.27 | -3.32 | <0.01* |
|  | 2012 - Pre-DWH | -0.99 | 0.28 | -3.58 | <0.01* |
|  | 2013 - Pre-DWH | -0.79 | 0.27 | -2.96 | 0.02* |
|  | 2014 - Pre-DWH | -0.72 | 0.31 | -2.3 | 0.12 |
|  | 2015 - Pre-DWH | -0.41 | 0.33 | -1.26 | 0.74 |
|  | 2016 - Pre-DWH | -1.45 | 0.34 | -4.22 | <0.01* |
|  | 2017 - Pre-DWH | -1.3 | 0.34 | -3.79 | <0.01* |
| Large Demersal Invertivore | 2010 - Pre-DWH | -1.17 | 0.4 | -2.88 | 0.03* |
|  | 2011 - Pre-DWH | -0.54 | 0.31 | -1.72 | 0.38 |
|  | 2012 - Pre-DWH | -0.73 | 0.33 | -2.17 | 0.16 |
|  | 2013 - Pre-DWH | -0.72 | 0.31 | -2.29 | 0.12 |
|  | 2014 - Pre-DWH | -0.62 | 0.39 | -1.57 | 0.48 |
|  | 2015 - Pre-DWH | -1.07 | 0.38 | -2.78 | 0.04* |
|  | 2016 - Pre-DWH | -0.85 | 0.41 | -2.08 | 0.2 |
|  | 2017 - Pre-DWH | -0.56 | 0.42 | -1.33 | 0.67 |
| Generalist Carnivore | 2010 - Pre-DWH | -0.98 | 0.35 | -2.77 | 0.04* |
|  | 2011 - Pre-DWH | -1.09 | 0.27 | -4.03 | <0.01* |
|  | 2012 - Pre-DWH | -0.98 | 0.28 | -3.58 | <0.01* |
|  | 2013 - Pre-DWH | -1.04 | 0.27 | -3.87 | <0.01* |
|  | 2014 - Pre-DWH | -1.6 | 0.3 | -5.27 | <0.01* |
|  | 2015 - Pre-DWH | -1.28 | 0.34 | -3.71 | <0.01* |
|  | 2016 - Pre-DWH | -1.97 | 0.34 | -5.72 | <0.01* |
|  | 2017 - Pre-DWH | -1.43 | 0.35 | -4.07 | <0.01* |
| Piscivore | 2010 - Pre-DWH | -1.1 | 0.26 | -4.2 | <0.01* |
|  | 2011 - Pre-DWH | -0.64 | 0.2 | -3.11 | 0.01* |
|  | 2012 - Pre-DWH | -0.8 | 0.21 | -3.81 | <0.01* |
|  | 2013 - Pre-DWH | -1 | 0.2 | -5.02 | <0.01* |
|  | 2014 - Pre-DWH | -1.07 | 0.24 | -4.49 | <0.01* |
|  | 2015 - Pre-DWH | -0.89 | 0.25 | -3.6 | <0.01* |
|  | 2016 - Pre-DWH | -0.98 | 0.27 | -3.7 | <0.01* |
|  | 2017 - Pre-DWH | -1.24 | 0.26 | -4.67 | <0.01* |
| Reef Planktivore | 2010 - Pre-DWH | -0.25 | 0.52 | -0.48 | 1 |
|  | 2011 - Pre-DWH | -0.78 | 0.38 | -2.05 | 0.22 |
|  | 2012 - Pre-DWH | -0.91 | 0.39 | -2.32 | 0.12 |
|  | 2013 - Pre-DWH | 0.59 | 0.38 | 1.54 | 0.52 |
|  | 2014 - Pre-DWH | 0.03 | 0.44 | 0.07 | 1 |
|  | 2015 - Pre-DWH | 1.21 | 0.47 | 2.57 | 0.06 |
|  | 2016 - Pre-DWH | 0.22 | 0.47 | 0.46 | 1 |
|  | 2017 - Pre-DWH | 0.42 | 0.49 | 0.85 | 0.95 |

**Table S5.** Results from the linear mixed effects model comparing species-specific presence/absence between pre- and post-DWH time points. For species not observed during the pre-DWH time bin, comparisons were made between the first and subsequent years with positive density estimates. A stroked t (ŧ) denote significant differences at α = 0.05.

| Trophic Guild | Scientific Name | Comparison | Estimate | SE | *z* | *P* |
| --- | --- | --- | --- | --- | --- | --- |
| Herbivore | *Acanthurus chirurgus* | 2010 - Pre-DWH | -1.57 | 1.34 | -1.17 | 0.8 |
|  |  | 2011 - Pre-DWH | -2.9 | 1.28 | -2.27 | 0.13 |
|  |  | 2012 - Pre-DWH | -1.17 | 0.94 | -1.25 | 0.74 |
|  |  | 2013 - Pre-DWH | -2.92 | 1.28 | -2.29 | 0.13 |
|  |  | 2014 - Pre-DWH | -0.53 | 0.99 | -0.54 | 1 |
|  |  | 2015 - Pre-DWH | -1.67 | 1.33 | -1.26 | 0.74 |
|  |  | 2016 - Pre-DWH | -- | -- | -- | -- |
|  |  | 2017 - Pre-DWH | -1.57 | 1.33 | -1.18 | 0.79 |
| Small Demersal Browser | *Canthigaster rostrata* | 2010 - Pre-DWH | -- | -- | -- | -- |
|  |  | 2011 - Pre-DWH | -0.42 | 0.73 | -0.57 | 0.99 |
|  |  | 2012 - Pre-DWH | -0.17 | 0.74 | -0.22 | 1 |
|  |  | 2013 - Pre-DWH | 1.51 | 0.67 | 2.26 | 0.12 |
|  |  | 2014 - Pre-DWH | 1.17 | 0.75 | 1.56 | 0.46 |
|  |  | 2015 - Pre-DWH | 3.27 | 0.94 | 3.49 | <0.01ŧ |
|  |  | 2016 - Pre-DWH | 0.86 | 0.85 | 1.01 | 0.85 |
|  |  | 2017 - Pre-DWH | 0.57 | 0.89 | 0.65 | 0.98 |
|  | *Chaetodon ocellatus* | 2010 - Pre-DWH | 0.11 | 1 | 0.11 | 1 |
|  |  | 2011 - Pre-DWH | -0.27 | 0.83 | -0.32 | 1 |
|  |  | 2012 - Pre-DWH | -0.08 | 0.63 | -0.13 | 1 |
|  |  | 2013 - Pre-DWH | 0.04 | 0.63 | 0.06 | 1 |
|  |  | 2014 - Pre-DWH | -0.15 | 0.62 | -0.25 | 1 |
|  |  | 2015 - Pre-DWH | 0.16 | 0.7 | 0.23 | 1 |
|  |  | 2016 - Pre-DWH | 0.77 | 0.75 | 1.03 | 0.87 |
|  |  | 2017 - Pre-DWH | -0.81 | 0.92 | -0.88 | 0.94 |

**Table S5 Continued**

| Trophic Guild | Scientific Name | Comparison | Estimate | SE | *z* | *P* |
| --- | --- | --- | --- | --- | --- | --- |
| Small Demersal Browser | *Chaetodon sedentarius* | 2010 - Pre-DWH | -0.02 | 0.84 | -0.03 | 1 |
|  |  | 2011 - Pre-DWH | 0.66 | 0.66 | 1.01 | 0.89 |
|  |  | 2012 - Pre-DWH | 0.57 | 0.68 | 0.84 | 0.96 |
|  |  | 2013 - Pre-DWH | 1.04 | 0.65 | 1.59 | 0.5 |
|  |  | 2014 - Pre-DWH | 0.83 | 0.76 | 1.09 | 0.85 |
|  |  | 2015 - Pre-DWH | 1.54 | 0.89 | 1.72 | 0.41 |
|  |  | 2016 - Pre-DWH | 0.57 | 0.85 | 0.66 | 0.99 |
|  |  | 2017 - Pre-DWH | -0.61 | 0.93 | -0.66 | 0.99 |
|  | *Parablennius marmoreus* | 2010 - Pre-DWH | -- | -- | -- | -- |
|  |  | 2011 - Pre-DWH | -0.06 | 0.57 | -0.1 | 1 |
|  |  | 2012 - Pre-DWH | -0.28 | 0.58 | -0.47 | 0.99 |
|  |  | 2013 - Pre-DWH | -0.91 | 0.62 | -1.47 | 0.51 |
|  |  | 2014 - Pre-DWH | -1.13 | 0.78 | -1.45 | 0.52 |
|  |  | 2015 - Pre-DWH | -- | -- | -- | -- |
|  |  | 2016 - Pre-DWH | -1.09 | 0.89 | -1.23 | 0.68 |
|  |  | 2017 - Pre-DWH | -1.02 | 0.9 | -1.14 | 0.74 |
|  | *Prognathodes aya* | 2010 - Pre-DWH | -1.71 | 1.22 | -1.4 | 0.6 |
|  |  | 2011 - Pre-DWH | 0.21 | 0.73 | 0.29 | 1 |
|  |  | 2012 - Pre-DWH | 0.15 | 0.76 | 0.2 | 1 |
|  |  | 2013 - Pre-DWH | 0.49 | 0.73 | 0.68 | 0.98 |
|  |  | 2014 - Pre-DWH | 0.17 | 0.84 | 0.2 | 1 |
|  |  | 2015 - Pre-DWH | 1.04 | 0.95 | 1.1 | 0.82 |
|  |  | 2016 - Pre-DWH | -- | -- | -- | -- |
|  |  | 2017 - Pre-DWH | 0.11 | 1 | 0.11 | 1 |
|  | *Stegastes leucostictus* | 2010 - Pre-DWH | -1.44 | 1.29 | -1.12 | 0.79 |
|  |  | 2011 - Pre-DWH | -0.65 | 0.82 | -0.79 | 0.96 |
|  |  | 2012 - Pre-DWH | -0.5 | 0.81 | -0.62 | 0.99 |
|  |  | 2013 - Pre-DWH | 1.2 | 0.76 | 1.58 | 0.46 |
|  |  | 2014 - Pre-DWH | 1.79 | 0.85 | 2.11 | 0.17 |
|  |  | 2015 - Pre-DWH | 2.02 | 0.94 | 2.16 | 0.16 |
|  |  | 2016 - Pre-DWH | 0.78 | 0.93 | 0.84 | 0.94 |
|  |  | 2017 - Pre-DWH | -1.42 | 0.56 | -2.55 | 0.06 |

**Table S5 Continued**

| Trophic Guild | Scientific Name | Comparison | Estimate | SE | *z* | *P* |
| --- | --- | --- | --- | --- | --- | --- |
| Small Demersal Browser | *Stegastes variabilis* | 2010 - Pre-DWH | -0.47 | 0.94 | -0.49 | 0.99 |
|  |  | 2011 - Pre-DWH | -1.53 | 0.81 | -1.88 | 0.26 |
|  |  | 2012 - Pre-DWH | -1.24 | 0.76 | -1.62 | 0.42 |
|  |  | 2013 - Pre-DWH | -1.25 | 0.76 | -1.65 | 0.4 |
|  |  | 2014 - Pre-DWH | -2.01 | 1.16 | -1.74 | 0.35 |
|  |  | 2015 - Pre-DWH | -- | -- | -- | -- |
|  |  | 2016 - Pre-DWH | -0.28 | 0.85 | -0.33 | 1 |
|  |  | 2017 - Pre-DWH | -- | -- | -- | -- |
| Large Demersal Browser | *Aluterus monoceros* | 2012 - 2011 | 1.35 | 0.98 | 1.38 | 0.5 |
|  |  | 2013 - 2011 | 0.91 | 0.99 | 0.92 | 0.83 |
|  |  | 2014 - 2011 | 1.81 | 1.12 | 1.62 | 0.35 |
|  |  | 2015 - 2011 | 0.51 | 1.4 | 0.36 | 1 |
|  |  | 2016 - 2011 | -- | -- | -- | -- |
|  |  | 2017 - 2011 | 0.53 | 1.4 | 0.38 | 1 |
|  | *Holacanthus bermudensis* | 2010 - Pre-DWH | 0.11 | 0.79 | 0.14 | 1 |
|  |  | 2011 - Pre-DWH | -0.04 | 0.61 | -0.06 | 1 |
|  |  | 2012 - Pre-DWH | -0.18 | 0.62 | -0.29 | 1 |
|  |  | 2013 - Pre-DWH | 0.66 | 0.62 | 1.05 | 0.87 |
|  |  | 2014 - Pre-DWH | -0.42 | 0.69 | -0.61 | 0.99 |
|  |  | 2015 - Pre-DWH | 0.35 | 0.81 | 0.43 | 1 |
|  |  | 2016 - Pre-DWH | 0.35 | 0.81 | 0.43 | 1 |
|  |  | 2017 - Pre-DWH | -0.59 | 0.8 | -0.73 | 0.98 |
|  | *Holacanthus ciliaris* | 2010 - Pre-DWH | -0.91 | 1.31 | -0.7 | 0.99 |
|  |  | 2011 - Pre-DWH | 0.36 | 0.83 | 0.43 | 1 |
|  |  | 2012 - Pre-DWH | -1.24 | 1.04 | -1.2 | 0.8 |
|  |  | 2013 - Pre-DWH | -0.6 | 0.9 | -0.67 | 0.99 |
|  |  | 2014 - Pre-DWH | -0.55 | 1.05 | -0.53 | 1 |
|  |  | 2015 - Pre-DWH | -0.94 | 1.29 | -0.73 | 0.98 |
|  |  | 2016 - Pre-DWH | -0.94 | 1.29 | -0.73 | 0.98 |
|  |  | 2017 - Pre-DWH | -0.88 | 1.3 | -0.68 | 0.99 |

**Table S5 Continued**

| Trophic Guild | Scientific Name | Comparison | Estimate | SE | *z* | *P* |
| --- | --- | --- | --- | --- | --- | --- |
| Small Demersal Invertivore | *Bodianus pulchellus* | 2010 - Pre-DWH | 0.53 | 0.85 | 0.62 | 0.99 |
|  |  | 2011 - Pre-DWH | -0.12 | 0.68 | -0.18 | 1 |
|  |  | 2012 - Pre-DWH | -0.6 | 0.72 | -0.83 | 0.96 |
|  |  | 2013 - Pre-DWH | 0.84 | 0.68 | 1.24 | 0.75 |
|  |  | 2014 - Pre-DWH | -0.31 | 0.82 | -0.38 | 1 |
|  |  | 2015 - Pre-DWH | 1.17 | 0.9 | 1.31 | 0.71 |
|  |  | 2016 - Pre-DWH | 0.68 | 0.89 | 0.77 | 0.97 |
|  |  | 2017 - Pre-DWH | -0.01 | 0.93 | -0.01 | 1 |
|  | Bodianus rufus | 2010 - Pre-DWH | -- | -- | -- | -- |
|  |  | 2011 - Pre-DWH | -1.24 | 1.55 | -0.8 | 0.91 |
|  |  | 2012 - Pre-DWH | 0.16 | 1.31 | 0.12 | 1 |
|  |  | 2013 - Pre-DWH | 0.03 | 1.31 | 0.02 | 1 |
|  |  | 2014 - Pre-DWH | 1.47 | 1.35 | 1.09 | 0.73 |
|  |  | 2015 - Pre-DWH | 1.01 | 1.44 | 0.7 | 0.95 |
|  |  | 2016 - Pre-DWH | 1.72 | 1.39 | 1.24 | 0.62 |
|  |  | 2017 - Pre-DWH | 0.06 | 1.61 | 0.04 | 1 |
|  | *Equetus lanceolatus* | 2010 - Pre-DWH | -0.27 | 1.05 | -0.26 | 1 |
|  |  | 2011 - Pre-DWH | -0.7 | 0.81 | -0.87 | 0.95 |
|  |  | 2012 - Pre-DWH | -1.44 | 0.88 | -1.63 | 0.47 |
|  |  | 2013 - Pre-DWH | 0.55 | 0.78 | 0.71 | 0.98 |
|  |  | 2014 - Pre-DWH | 0.67 | 0.89 | 0.75 | 0.98 |
|  |  | 2015 - Pre-DWH | 0.76 | 1.02 | 0.75 | 0.98 |
|  |  | 2016 - Pre-DWH | 0.12 | 1.02 | 0.12 | 1 |
|  |  | 2017 - Pre-DWH | 2.14 | 1.08 | 1.98 | 0.25 |
|  | *Haemulon aurolineatum* | 2010 - Pre-DWH | -0.78 | 0.75 | -1.05 | 0.87 |
|  |  | 2011 - Pre-DWH | -0.52 | 0.58 | -0.9 | 0.94 |
|  |  | 2012 - Pre-DWH | 0.02 | 0.59 | 0.03 | 1 |
|  |  | 2013 - Pre-DWH | 0.16 | 0.58 | 0.28 | 1 |
|  |  | 2014 - Pre-DWH | 0.29 | 0.67 | 0.44 | 1 |
|  |  | 2015 - Pre-DWH | 0.67 | 0.79 | 0.85 | 0.95 |
|  |  | 2016 - Pre-DWH | 0.67 | 0.79 | 0.85 | 0.95 |
|  |  | 2017 - Pre-DWH | 1.55 | 0.9 | 1.71 | 0.41 |

**Table S5 Continued**

| Trophic Guild | Scientific Name | Comparison | Estimate | SE | *z* | *P* |
| --- | --- | --- | --- | --- | --- | --- |
| Small Demersal Invertivore | *Halichoeres bivittatus* | 2010 - Pre-DWH | -3.06 | 1.13 | -2.72 | 0.04ŧ |
|  |  | 2011 - Pre-DWH | -1.19 | 0.54 | -2.22 | 0.15 |
|  |  | 2012 - Pre-DWH | -1.51 | 0.55 | -2.73 | 0.04ŧ |
|  |  | 2013 - Pre-DWH | -1.94 | 0.57 | -3.42 | <0.01ŧ |
|  |  | 2014 - Pre-DWH | -1.93 | 0.67 | -2.9 | 0.03ŧ |
|  |  | 2015 - Pre-DWH | 0.03 | 0.69 | 0.04 | 1 |
|  |  | 2016 - Pre-DWH | -0.79 | 0.68 | -1.17 | 0.81 |
|  |  | 2017 - Pre-DWH | -0.67 | 0.69 | -0.98 | 0.91 |
|  | *Holocentrus adscensionis* | 2010 - Pre-DWH | -- | -- | -- | -- |
|  |  | 2011 - Pre-DWH | -0.39 | 0.82 | -0.47 | 1 |
|  |  | 2012 - Pre-DWH | 0.16 | 0.79 | 0.2 | 1 |
|  |  | 2013 - Pre-DWH | 0.34 | 0.76 | 0.45 | 1 |
|  |  | 2014 - Pre-DWH | -1.23 | 1.22 | -1 | 0.86 |
|  |  | 2015 - Pre-DWH | 1.14 | 0.88 | 1.3 | 0.66 |
|  |  | 2016 - Pre-DWH | -1.01 | 1.24 | -0.81 | 0.94 |
|  |  | 2017 - Pre-DWH | -0.9 | 1.25 | -0.72 | 0.97 |
|  | *Holocentrus rufus* | 2012 - 2011 | -0.74 | 0.94 | -0.79 | 0.87 |
|  |  | 2013 - 2011 | -- | -- | -- | -- |
|  |  | 2014 - 2011 | 0.8 | 0.9 | 0.89 | 0.82 |
|  |  | 2015 - 2011 | -- | -- | -- | -- |
|  |  | 2016 - 2011 | 0.51 | 1 | 0.51 | 0.97 |
|  |  | 2017 - 2011 | 0.76 | 1.02 | 0.75 | 0.89 |
|  | *Pareques acuminatus* | 2010 - Pre-DWH | -- | -- | -- | -- |
|  |  | 2011 - Pre-DWH | -1.52 | 0.93 | -1.62 | 0.38 |
|  |  | 2012 - Pre-DWH | -1.54 | 0.94 | -1.64 | 0.37 |
|  |  | 2013 - Pre-DWH | -2.28 | 1.17 | -1.94 | 0.21 |
|  |  | 2014 - Pre-DWH | -1 | 0.95 | -1.05 | 0.79 |
|  |  | 2015 - Pre-DWH | -- | -- | -- | -- |
|  |  | 2016 - Pre-DWH | -0.3 | 0.97 | -0.31 | 1 |
|  |  | 2017 - Pre-DWH | -- | -- | -- | -- |

**Table S5 Continued**

| Trophic Guild | Scientific Name | Comparison | Estimate | SE | *z* | *P* |
| --- | --- | --- | --- | --- | --- | --- |
| Small Demersal Invertivore | *Pareques umbrosus* | 2010 - Pre-DWH | -0.3 | 0.71 | -0.42 | 1 |
|  |  | 2011 - Pre-DWH | -0.07 | 0.55 | -0.13 | 1 |
|  |  | 2012 - Pre-DWH | -0.51 | 0.56 | -0.91 | 0.93 |
|  |  | 2013 - Pre-DWH | -0.16 | 0.54 | -0.29 | 1 |
|  |  | 2014 - Pre-DWH | 0.48 | 0.62 | 0.77 | 0.97 |
|  |  | 2015 - Pre-DWH | -0.06 | 0.7 | -0.08 | 1 |
|  |  | 2016 - Pre-DWH | -0.06 | 0.7 | -0.08 | 1 |
|  |  | 2017 - Pre-DWH | -0.52 | 0.73 | -0.7 | 0.98 |
| Large Demersal Invertivore | *Archosargus probatocephalus* | 2010 - Pre-DWH | -- | -- | -- | -- |
|  |  | 2011 - Pre-DWH | -0.3 | 1.46 | -0.2 | 1 |
|  |  | 2012 - Pre-DWH | 1.36 | 1.32 | 1.03 | 0.73 |
|  |  | 2013 - Pre-DWH | 0.13 | 1.4 | 0.1 | 1 |
|  |  | 2014 - Pre-DWH | 0.82 | 1.44 | 0.57 | 0.97 |
|  |  | 2015 - Pre-DWH | 0.13 | 1.72 | 0.08 | 1 |
|  |  | 2016 - Pre-DWH | -- | -- | -- | -- |
|  |  | 2017 - Pre-DWH | 0.13 | 1.72 | 0.08 | 1 |
|  | *Balistes capriscus* | 2010 - Pre-DWH | -0.32 | 0.84 | -0.38 | 1 |
|  |  | 2011 - Pre-DWH | -0.6 | 0.63 | -0.94 | 0.91 |
|  |  | 2012 - Pre-DWH | -0.53 | 0.63 | -0.83 | 0.95 |
|  |  | 2013 - Pre-DWH | 0.14 | 0.63 | 0.22 | 1 |
|  |  | 2014 - Pre-DWH | -0.47 | 0.71 | -0.67 | 0.99 |
|  |  | 2015 - Pre-DWH | 0.67 | 0.82 | 0.81 | 0.96 |
|  |  | 2016 - Pre-DWH | 0.67 | 0.82 | 0.81 | 0.96 |
|  |  | 2017 - Pre-DWH | 1.49 | 0.9 | 1.65 | 0.43 |
|  | *Chilomycterus schoepfi* | 2012 - 2011 | -0.29 | 0.82 | -0.36 | 1 |
|  |  | 2013 - 2011 | -- | -- | -- | -- |
|  |  | 2014 - 2011 | 0.54 | 0.85 | 0.64 | 0.97 |
|  |  | 2015 - 2011 | 0.45 | 0.96 | 0.47 | 0.99 |
|  |  | 2016 - 2011 | 0.98 | 0.87 | 1.12 | 0.73 |
|  |  | 2017 - 2011 | -0.29 | 1.2 | -0.25 | 1 |

**Table S5 Continued**

| Trophic Guild | Scientific Name | Comparison | Estimate | SE | *z* | *P* |
| --- | --- | --- | --- | --- | --- | --- |
| Large Demersal Invertivore | *Pagrus pagrus* | 2010 - Pre-DWH | 0.12 | 0.77 | 0.15 | 1 |
|  |  | 2011 - Pre-DWH | 1.44 | 0.62 | 2.3 | 0.12 |
|  |  | 2012 - Pre-DWH | 0.05 | 0.62 | 0.08 | 1 |
|  |  | 2013 - Pre-DWH | 0.25 | 0.6 | 0.42 | 1 |
|  |  | 2014 - Pre-DWH | 0.39 | 0.68 | 0.57 | 0.99 |
|  |  | 2015 - Pre-DWH | -0.61 | 0.81 | -0.76 | 0.97 |
|  |  | 2016 - Pre-DWH | -0.22 | 0.78 | -0.28 | 1 |
|  |  | 2017 - Pre-DWH | 0.26 | 0.78 | 0.33 | 1 |
| Generalist Carnivore | *Caranx crysos* | 2010 - Pre-DWH | -0.7 | 1.24 | -0.56 | 0.99 |
|  |  | 2011 - Pre-DWH | -1.17 | 0.99 | -1.18 | 0.78 |
|  |  | 2012 - Pre-DWH | 0.06 | 0.81 | 0.08 | 1 |
|  |  | 2013 - Pre-DWH | -1.22 | 0.99 | -1.23 | 0.74 |
|  |  | 2014 - Pre-DWH | -1.24 | 1.22 | -1.01 | 0.88 |
|  |  | 2015 - Pre-DWH | 0.55 | 0.95 | 0.58 | 0.99 |
|  |  | 2016 - Pre-DWH | -- | -- | -- | -- |
|  |  | 2017 - Pre-DWH | -0.73 | 1.25 | -0.59 | 0.99 |
|  | *Centropristis ocyurus* | 2010 - Pre-DWH | -1.75 | 1.14 | -1.55 | 0.53 |
|  |  | 2011 - Pre-DWH | 0.13 | 0.59 | 0.22 | 1 |
|  |  | 2012 - Pre-DWH | -0.44 | 0.61 | -0.73 | 0.98 |
|  |  | 2013 - Pre-DWH | -0.17 | 0.59 | -0.29 | 1 |
|  |  | 2014 - Pre-DWH | 0.4 | 0.66 | 0.6 | 0.99 |
|  |  | 2015 - Pre-DWH | 0.52 | 0.74 | 0.7 | 0.99 |
|  |  | 2016 - Pre-DWH | -0.56 | 0.79 | -0.7 | 0.98 |
|  |  | 2017 - Pre-DWH | -1.45 | 0.94 | -1.53 | 0.54 |
|  | *Diplectrum formosum* | 2012 - 2011 | 1.47 | 1.16 | 1.27 | 0.44 |
|  |  | 2013 - 2011 | 1.74 | 1.14 | 1.53 | 0.29 |
|  |  | 2014 - 2011 | 0.59 | 1.46 | 0.41 | 0.97 |
|  |  | 2015 - 2011 | 2.93 | 1.22 | 2.41 | 0.05ŧ |

**Table S5 Continued**

| Trophic Guild | Scientific Name | Comparison | Estimate | SE | *z* | *P* |
| --- | --- | --- | --- | --- | --- | --- |
| Generalist Carnivore | *Epinephelus cruentatus* | 2010 - Pre-DWH | -- | -- | -- | -- |
|  |  | 2011 - Pre-DWH | -0.57 | 0.98 | -0.58 | 0.99 |
|  |  | 2012 - Pre-DWH | 0.3 | 0.93 | 0.32 | 1 |
|  |  | 2013 - Pre-DWH | 0.57 | 0.88 | 0.65 | 0.98 |
|  |  | 2014 - Pre-DWH | 0.83 | 1 | 0.83 | 0.93 |
|  |  | 2015 - Pre-DWH | 1.67 | 1.08 | 1.54 | 0.46 |
|  |  | 2016 - Pre-DWH | 0.32 | 1.16 | 0.27 | 1 |
|  |  | 2017 - Pre-DWH | 1.71 | 1.09 | 1.57 | 0.44 |
|  | *Epinephelus morio* | 2010 - Pre-DWH | -0.23 | 0.74 | -0.31 | 1 |
|  |  | 2011 - Pre-DWH | -1.16 | 0.6 | -1.92 | 0.28 |
|  |  | 2012 - Pre-DWH | -1.36 | 0.64 | -2.13 | 0.18 |
|  |  | 2013 - Pre-DWH | -1.18 | 0.6 | -1.97 | 0.25 |
|  |  | 2014 - Pre-DWH | -1.61 | 0.78 | -2.07 | 0.2 |
|  |  | 2015 - Pre-DWH | -1.18 | 0.81 | -1.46 | 0.58 |
|  |  | 2016 - Pre-DWH | -2.52 | 1.14 | -2.2 | 0.15 |
|  |  | 2017 - Pre-DWH | -- | -- | -- | -- |
|  | *Liopropoma eukrines* | 2010 - Pre-DWH | 1.2 | 0.95 | 1.26 | 0.71 |
|  |  | 2011 - Pre-DWH | 1.19 | 0.79 | 1.5 | 0.53 |
|  |  | 2012 - Pre-DWH | 2.09 | 0.82 | 2.54 | 0.07 |
|  |  | 2013 - Pre-DWH | 1.73 | 0.79 | 2.2 | 0.15 |
|  |  | 2014 - Pre-DWH | 2.32 | 0.91 | 2.56 | 0.06 |
|  |  | 2015 - Pre-DWH | 3.56 | 1.05 | 3.39 | <0.01ŧ |
|  |  | 2016 - Pre-DWH | 0.74 | 1.03 | 0.71 | 0.98 |
|  |  | 2017 - Pre-DWH | 1.03 | 1.06 | 0.97 | 0.9 |
|  | *Lutjanus campechanus* | 2010 - Pre-DWH | -0.98 | 1.13 | -0.87 | 0.91 |
|  |  | 2011 - Pre-DWH | -0.52 | 0.94 | -0.55 | 0.99 |
|  |  | 2012 - Pre-DWH | -1.53 | 0.9 | -1.7 | 0.36 |
|  |  | 2013 - Pre-DWH | -0.88 | 0.9 | -0.97 | 0.86 |
|  |  | 2014 - Pre-DWH | -1.09 | 0.99 | -1.11 | 0.78 |
|  |  | 2015 - Pre-DWH | -1.71 | 1.03 | -1.66 | 0.38 |
|  |  | 2016 - Pre-DWH | -1.24 | 1.06 | -1.17 | 0.73 |
|  |  | 2017 - Pre-DWH | 0.12 | 1.34 | 0.09 | 1 |

**Table S5 Continued**

| Trophic Guild | Scientific Name | Comparison | Estimate | SE | *z* | *P* |
| --- | --- | --- | --- | --- | --- | --- |
| Generalist Carnivore | *Lutjanus griseus* | 2010 - Pre-DWH | -1.03 | 0.93 | -1.1 | 0.81 |
|  |  | 2011 - Pre-DWH | -0.79 | 0.69 | -1.13 | 0.79 |
|  |  | 2012 - Pre-DWH | -0.94 | 0.7 | -1.34 | 0.64 |
|  |  | 2013 - Pre-DWH | -1.39 | 0.7 | -1.98 | 0.23 |
|  |  | 2014 - Pre-DWH | -1.33 | 0.79 | -1.69 | 0.39 |
|  |  | 2015 - Pre-DWH | -0.81 | 0.86 | -0.94 | 0.9 |
|  |  | 2016 - Pre-DWH | -1.24 | 0.88 | -1.41 | 0.58 |
|  |  | 2017 - Pre-DWH | -0.31 | 0.87 | -0.35 | 1 |
|  | *Lutjanus synagris* | 2010 - Pre-DWH | -1.38 | 0.93 | -1.48 | 0.59 |
|  |  | 2011 - Pre-DWH | -2.72 | 0.89 | -3.07 | 0.02ŧ |
|  |  | 2012 - Pre-DWH | -0.73 | 0.62 | -1.18 | 0.81 |
|  |  | 2013 - Pre-DWH | -1.15 | 0.64 | -1.8 | 0.36 |
|  |  | 2014 - Pre-DWH | -1.22 | 0.75 | -1.62 | 0.49 |
|  |  | 2015 - Pre-DWH | -0.58 | 0.78 | -0.75 | 0.98 |
|  |  | 2016 - Pre-DWH | -1.52 | 0.92 | -1.65 | 0.47 |
|  |  | 2017 - Pre-DWH | -0.15 | 0.76 | -0.2 | 1 |
|  | *Pterois volitans* | 2012 - 2011 | 3.64 | 1.12 | 3.25 | <0.01ŧ |
|  |  | 2013 - 2011 | 5.2 | 1.14 | 4.55 | <0.01ŧ |
|  |  | 2014 - 2011 | 4.93 | 1.2 | 4.12 | <0.01ŧ |
|  |  | 2015 - 2011 | 6.44 | 1.34 | 4.79 | <0.01ŧ |
|  |  | 2016 - 2011 | 5.94 | 1.3 | 4.55 | <0.01ŧ |
|  |  | 2017 - 2011 | 6.37 | 1.35 | 4.71 | <0.01ŧ |
|  | *Rypticus maculatus* | 2010 - Pre-DWH | -1.05 | 1 | -1.06 | 0.85 |
|  |  | 2011 - Pre-DWH | 0.54 | 0.64 | 0.83 | 0.95 |
|  |  | 2012 - Pre-DWH | -0.05 | 0.66 | -0.07 | 1 |
|  |  | 2013 - Pre-DWH | 0.03 | 0.65 | 0.05 | 1 |
|  |  | 2014 - Pre-DWH | 0.31 | 0.72 | 0.43 | 1 |
|  |  | 2015 - Pre-DWH | 0.98 | 0.79 | 1.24 | 0.73 |
|  |  | 2016 - Pre-DWH | -1.61 | 1.02 | -1.58 | 0.47 |
|  |  | 2017 - Pre-DWH | 0.11 | 0.83 | 0.13 | 1 |

**Table S5 Continued**

| Trophic Guild | Scientific Name | Comparison | Estimate | SE | *z* | *P* |
| --- | --- | --- | --- | --- | --- | --- |
| Generalist Carnivore | *Seriola dumerili* | 2010 - Pre-DWH | -0.08 | 0.83 | -0.1 | 1 |
|  |  | 2011 - Pre-DWH | -1.35 | 0.62 | -2.18 | 0.16 |
|  |  | 2012 - Pre-DWH | -0.81 | 0.63 | -1.3 | 0.7 |
|  |  | 2013 - Pre-DWH | -1.09 | 0.61 | -1.78 | 0.35 |
|  |  | 2014 - Pre-DWH | -1.48 | 0.7 | -2.12 | 0.18 |
|  |  | 2015 - Pre-DWH | 0.63 | 0.85 | 0.74 | 0.98 |
|  |  | 2016 - Pre-DWH | -0.97 | 0.78 | -1.24 | 0.74 |
|  |  | 2017 - Pre-DWH | -0.26 | 0.8 | -0.33 | 1 |
|  | *Seriola rivoliana* | 2010 - Pre-DWH | -- | -- | -- | -- |
|  |  | 2011 - Pre-DWH | -1.86 | 0.68 | -2.75 | 0.04ŧ |
|  |  | 2012 - Pre-DWH | -1.36 | 0.65 | -2.1 | 0.18 |
|  |  | 2013 - Pre-DWH | -1.36 | 0.63 | -2.18 | 0.15 |
|  |  | 2014 - Pre-DWH | -1.73 | 0.8 | -2.16 | 0.16 |
|  |  | 2015 - Pre-DWH | -0.23 | 0.74 | -0.32 | 1 |
|  |  | 2016 - Pre-DWH | -1.36 | 0.84 | -1.63 | 0.44 |
|  |  | 2017 - Pre-DWH | -1.24 | 0.85 | -1.47 | 0.55 |
|  | *Serranus phoebe* | 2010 - Pre-DWH | -0.56 | 0.96 | -0.58 | 0.99 |
|  |  | 2011 - Pre-DWH | 0.13 | 0.66 | 0.2 | 1 |
|  |  | 2012 - Pre-DWH | 0.38 | 0.67 | 0.56 | 1 |
|  |  | 2013 - Pre-DWH | 0.05 | 0.66 | 0.08 | 1 |
|  |  | 2014 - Pre-DWH | 1.33 | 0.73 | 1.81 | 0.33 |
|  |  | 2015 - Pre-DWH | 1.65 | 0.82 | 2 | 0.23 |
|  |  | 2016 - Pre-DWH | 0.48 | 0.83 | 0.57 | 1 |
|  |  | 2017 - Pre-DWH | 1.17 | 0.83 | 1.4 | 0.62 |
|  | *Serranus subligarius* | 2010 - Pre-DWH | -- | -- | -- | -- |
|  |  | 2011 - Pre-DWH | -0.56 | 0.74 | -0.76 | 0.97 |
|  |  | 2012 - Pre-DWH | -0.83 | 0.75 | -1.11 | 0.83 |
|  |  | 2013 - Pre-DWH | -2.07 | 0.89 | -2.33 | 0.11 |
|  |  | 2014 - Pre-DWH | -2.81 | 1.24 | -2.26 | 0.13 |
|  |  | 2015 - Pre-DWH | -1.14 | 1.04 | -1.1 | 0.84 |
|  |  | 2016 - Pre-DWH | -2.07 | 1.27 | -1.63 | 0.46 |
|  |  | 2017 - Pre-DWH | -2.04 | 1.28 | -1.6 | 0.48 |

**Table S5 Continued**

| Trophic Guild | Scientific Name | Comparison | Estimate | SE | *z* | *P* |
| --- | --- | --- | --- | --- | --- | --- |
| Piscivore | *Carcharhinus plumbeus* | 2010 - Pre-DWH | -- | -- | -- | -- |
|  |  | 2011 - Pre-DWH | -1.89 | 1.19 | -1.59 | 0.5 |
|  |  | 2012 - Pre-DWH | -1.15 | 0.97 | -1.19 | 0.79 |
|  |  | 2013 - Pre-DWH | -0.48 | 0.82 | -0.58 | 0.99 |
|  |  | 2014 - Pre-DWH | -1.2 | 1.2 | -1 | 0.89 |
|  |  | 2015 - Pre-DWH | -0.79 | 1.21 | -0.65 | 0.99 |
|  |  | 2016 - Pre-DWH | -0.02 | 0.99 | -0.02 | 1 |
|  |  | 2017 - Pre-DWH | -0.72 | 1.22 | -0.59 | 0.99 |
|  | *Mycteroperca microlepis* | 2010 - Pre-DWH | -1.09 | 0.9 | -1.22 | 0.8 |
|  |  | 2011 - Pre-DWH | -1.49 | 0.68 | -2.19 | 0.17 |
|  |  | 2012 - Pre-DWH | -0.52 | 0.6 | -0.87 | 0.96 |
|  |  | 2013 - Pre-DWH | -2.11 | 0.77 | -2.73 | 0.04ŧ |
|  |  | 2014 - Pre-DWH | -0.33 | 0.66 | -0.49 | 1 |
|  |  | 2015 - Pre-DWH | -0.41 | 0.76 | -0.54 | 1 |
|  |  | 2016 - Pre-DWH | -0.81 | 0.81 | -1 | 0.92 |
|  |  | 2017 - Pre-DWH | -1.99 | 1.15 | -1.73 | 0.42 |
|  | *Mycteroperca phenax* | 2010 - Pre-DWH | -0.88 | 0.84 | -1.04 | 0.84 |
|  |  | 2011 - Pre-DWH | 0.28 | 0.69 | 0.41 | 1 |
|  |  | 2012 - Pre-DWH | -0.37 | 0.68 | -0.54 | 0.99 |
|  |  | 2013 - Pre-DWH | -0.45 | 0.67 | -0.68 | 0.98 |
|  |  | 2014 - Pre-DWH | -1.05 | 0.74 | -1.42 | 0.57 |
|  |  | 2015 - Pre-DWH | 0.05 | 0.84 | 0.06 | 1 |
|  |  | 2016 - Pre-DWH | -1.05 | 0.82 | -1.29 | 0.67 |
|  |  | 2017 - Pre-DWH | 0.01 | 0.85 | 0.01 | 1 |
| Reef Planktivore | *Apogon pseudomaculatus* | 2010 - Pre-DWH | -0.05 | 1.27 | -0.04 | 1 |
|  |  | 2011 - Pre-DWH | 0.83 | 0.85 | 0.97 | 0.84 |
|  |  | 2012 - Pre-DWH | 0.52 | 0.89 | 0.58 | 0.99 |
|  |  | 2013 - Pre-DWH | 0.14 | 0.91 | 0.15 | 1 |
|  |  | 2014 - Pre-DWH | 0.9 | 0.92 | 0.97 | 0.84 |
|  |  | 2015 - Pre-DWH | 1.08 | 0.98 | 1.09 | 0.76 |
|  |  | 2016 - Pre-DWH | -0.17 | 1.27 | -0.13 | 1 |
|  |  | 2017 - Pre-DWH | -- | -- | -- | -- |

**Table S5 Continued**

| Trophic Guild | Scientific Name | Comparison | Estimate | SE | *z* | *P* |
| --- | --- | --- | --- | --- | --- | --- |
| Reef Planktivore | *Baldwinella aureorubens* | 2012 - 2011 | -- | -- | -- | -- |
|  |  | 2013 - 2011 | 0.41 | 0.97 | 0.42 | 0.99 |
|  |  | 2014 - 2011 | -0.32 | 1.37 | -0.24 | 1 |
|  |  | 2015 - 2011 | 2.85 | 1.23 | 2.32 | 0.09 |
|  |  | 2016 - 2011 | -0.05 | 1.43 | -0.04 | 1 |
|  |  | 2017 - 2011 | 2.26 | 1.26 | 1.79 | 0.28 |
|  | *Chromis enchrysura* | 2010 - Pre-DWH | -2.94 | 0.99 | -2.95 | 0.02* |
|  |  | 2011 - Pre-DWH | -1.01 | 0.67 | -1.5 | 0.57 |
|  |  | 2012 - Pre-DWH | -0.93 | 0.69 | -1.34 | 0.69 |
|  |  | 2013 - Pre-DWH | -0.13 | 0.67 | -0.2 | 1 |
|  |  | 2014 - Pre-DWH | 0.28 | 0.79 | 0.36 | 1 |
|  |  | 2015 - Pre-DWH | 2.56 | 1.13 | 2.26 | 0.14 |
|  |  | 2016 - Pre-DWH | 0.03 | 0.89 | 0.04 | 1 |
|  |  | 2017 - Pre-DWH | -1.05 | 0.88 | -1.18 | 0.8 |
|  | *Paranthias furcifer* | 2010 - Pre-DWH | -0.26 | 0.85 | -0.31 | 1 |
|  |  | 2011 - Pre-DWH | 1.55 | 0.76 | 2.04 | 0.21 |
|  |  | 2012 - Pre-DWH | 1.48 | 0.77 | 1.91 | 0.27 |
|  |  | 2013 - Pre-DWH | 2.03 | 0.78 | 2.6 | 0.05 |
|  |  | 2014 - Pre-DWH | 0.27 | 0.79 | 0.34 | 1 |
|  |  | 2015 - Pre-DWH | -3.19 | 1.27 | -2.51 | 0.07 |
|  |  | 2016 - Pre-DWH | -- | -- | -- | -- |
|  |  | 2017 - Pre-DWH | -1.43 | 0.96 | -1.49 | 0.54 |
|  | *Priacanthus arenatus* | 2010 - Pre-DWH | -0.26 | 0.85 | -0.31 | 1 |
|  |  | 2011 - Pre-DWH | 1.55 | 0.76 | 2.04 | 0.21 |
|  |  | 2012 - Pre-DWH | 1.48 | 0.77 | 1.91 | 0.27 |
|  |  | 2013 - Pre-DWH | 2.03 | 0.78 | 2.6 | 0.05 |
|  |  | 2014 - Pre-DWH | 0.27 | 0.79 | 0.34 | 1 |
|  |  | 2015 - Pre-DWH | -3.19 | 1.27 | -2.51 | 0.07 |
|  |  | 2016 - Pre-DWH | -- | -- | -- | -- |
|  |  | 2017 - Pre-DWH | -1.43 | 0.96 | -1.49 | 0.54 |

**Table S5 Continued**

| Trophic Guild | Scientific Name | Comparison | Estimate | SE | *z* | *P* |
| --- | --- | --- | --- | --- | --- | --- |
| Reef Planktivore | *Pristigenys alta* | 2010 - Pre-DWH | -- | -- | -- | -- |
|  |  | 2011 - Pre-DWH | -3.12 | 1.15 | -2.72 | 0.04ŧ |
|  |  | 2012 - Pre-DWH | -- | -- | -- | -- |
|  |  | 2013 - Pre-DWH | -3.14 | 1.14 | -2.74 | 0.03* |
|  |  | 2014 - Pre-DWH | -0.26 | 0.85 | -0.3 | 1 |
|  |  | 2015 - Pre-DWH | 4.25 | 1.34 | 3.17 | <0.01ŧ |
|  |  | 2016 - Pre-DWH | 2.79 | 1.04 | 2.68 | 0.04ŧ |
|  |  | 2017 - Pre-DWH | 2.78 | 1.04 | 2.66 | 0.04ŧ |
|  | *Pronotogrammus martinicensis* | 2010 - Pre-DWH | 0.02 | 1.19 | 0.02 | 1 |
|  |  | 2011 - Pre-DWH | 1.16 | 1.09 | 1.07 | 0.85 |
|  |  | 2012 - Pre-DWH | 1.03 | 1.11 | 0.92 | 0.93 |
|  |  | 2013 - Pre-DWH | 2.26 | 1.16 | 1.95 | 0.26 |
|  |  | 2014 - Pre-DWH | 2.38 | 1.36 | 1.75 | 0.38 |
|  |  | 2015 - Pre-DWH | 3.2 | 1.6 | 2 | 0.24 |
|  |  | 2016 - Pre-DWH | 0.71 | 1.34 | 0.53 | 1 |
|  |  | 2017 - Pre-DWH | 1.54 | 1.51 | 1.02 | 0.88 |
|  | *Rhomboplites aurorubens* | 2010 - Pre-DWH | 0.31 | 0.95 | 0.32 | 1 |
|  |  | 2011 - Pre-DWH | -0.1 | 0.68 | -0.15 | 1 |
|  |  | 2012 - Pre-DWH | 0.29 | 0.69 | 0.41 | 1 |
|  |  | 2013 - Pre-DWH | 0.38 | 0.68 | 0.56 | 1 |
|  |  | 2014 - Pre-DWH | 0.17 | 0.76 | 0.22 | 1 |
|  |  | 2015 - Pre-DWH | 2.35 | 1.08 | 2.17 | 0.17 |
|  |  | 2016 - Pre-DWH | 2.35 | 1.08 | 2.17 | 0.17 |
|  |  | 2017 - Pre-DWH | 1.59 | 0.99 | 1.6 | 0.48 |
|  | *Stegastes partitus* | 2010 - Pre-DWH | -1.32 | 1.43 | -0.93 | 0.84 |
|  |  | 2011 - Pre-DWH | -- | -- | -- | -- |
|  |  | 2012 - Pre-DWH | -- | -- | -- | -- |
|  |  | 2013 - Pre-DWH | -1.81 | 1.16 | -1.56 | 0.41 |
|  |  | 2014 - Pre-DWH | -0.67 | 1.17 | -0.57 | 0.98 |
|  |  | 2015 - Pre-DWH | 0.52 | 1.17 | 0.44 | 0.99 |
|  |  | 2016 - Pre-DWH | -1.28 | 1.44 | -0.89 | 0.86 |
|  |  | 2017 - Pre-DWH | -- | -- | -- | -- |

**Table S5 Continued**

| Trophic Guild | Scientific Name | Comparison | Estimate | SE | *z* | *P* |
| --- | --- | --- | --- | --- | --- | --- |
| Reef Planktivore | Damselfish | 2010 - Pre-DWH | -2.1 | 1.08 | -1.95 | 0.26 |
|  |  | 2011 - Pre-DWH | -1.33 | 0.78 | -1.72 | 0.39 |
|  |  | 2012 - Pre-DWH | -2.44 | 0.87 | -2.8 | 0.03ŧ |
|  |  | 2013 - Pre-DWH | 0.71 | 0.74 | 0.95 | 0.91 |
|  |  | 2014 - Pre-DWH | 0.06 | 0.85 | 0.07 | 1 |
|  |  | 2015 - Pre-DWH | 2.42 | 0.98 | 2.47 | 0.08 |
|  |  | 2016 - Pre-DWH | -1.41 | 0.99 | -1.42 | 0.6 |
|  |  | 2017 - Pre-DWH | 0.72 | 0.95 | 0.76 | 0.97 |

**Table S6.** Results from the linear mixed effects model comparing species-specific abundance estimates between pre- and post-DWH time points. For species not observed during the pre-DWH time bin, comparisons were made between the first and subsequent years with positive density estimates. Asterisks (*) denote significant differences at α = 0.05.

| Trophic Guild | Scientific Name | Comparison | Estimate | SE | *z* | *P* |
| --- | --- | --- | --- | --- | --- | --- |
| Herbivore | *Acanthurus chirurgus* | 2010 - Pre-DWH | -1.71 | 0.46 | -3.72 | <0.01* |
|  |  | 2011 - Pre-DWH | 1.67 | 0.81 | 2.07 | 0.2 |
|  |  | 2012 - Pre-DWH | -0.19 | 0.43 | -0.44 | 1 |
|  |  | 2013 - Pre-DWH | 0.58 | 0.53 | 1.11 | 0.82 |
|  |  | 2014 - Pre-DWH | -0.47 | 0.41 | -1.16 | 0.79 |
|  |  | 2015 - Pre-DWH | -1.38 | 0.46 | -3 | 0.02* |
|  |  | 2016 - Pre-DWH | -- | -- | -- | -- |
|  |  | 2017 - Pre-DWH | -1.38 | 0.46 | -3 | 0.02* |
| Small Demersal Browser | *Canthigaster rostrata* | 2010 - Pre-DWH | -- | -- | -- | -- |
|  |  | 2011 - Pre-DWH | -0.88 | 0.28 | -3.16 | <0.01* |
|  |  | 2012 - Pre-DWH | -0.82 | 0.28 | -2.87 | 0.02* |
|  |  | 2013 - Pre-DWH | -0.82 | 0.25 | -3.33 | <0.01* |
|  |  | 2014 - Pre-DWH | -0.91 | 0.27 | -3.41 | <0.01* |
|  |  | 2015 - Pre-DWH | -0.53 | 0.25 | -2.12 | 0.14 |
|  |  | 2016 - Pre-DWH | -1.29 | 0.3 | -4.3 | <0.01* |
|  |  | 2017 - Pre-DWH | -1.04 | 0.32 | -3.25 | <0.01* |
|  | *Chaetodon ocellatus* | 2010 - Pre-DWH | -1.14 | 0.28 | -4.09 | <0.01* |
|  |  | 2011 - Pre-DWH | -0.66 | 0.21 | -3.1 | 0.01* |
|  |  | 2012 - Pre-DWH | -0.44 | 0.22 | -2.02 | 0.22 |
|  |  | 2013 - Pre-DWH | -0.72 | 0.21 | -3.38 | <0.01* |
|  |  | 2014 - Pre-DWH | -1.03 | 0.23 | -4.41 | <0.01* |
|  |  | 2015 - Pre-DWH | -1.02 | 0.23 | -4.48 | <0.01* |
|  |  | 2016 - Pre-DWH | -0.99 | 0.33 | -2.95 | 0.02* |
|  |  | 2017 - Pre-DWH | -0.51 | 0.24 | -2.09 | 0.19 |

**Table S6 Continued**

| Trophic Guild | Scientific Name | Comparison | Estimate | SE | *z* | *P* |
| --- | --- | --- | --- | --- | --- | --- |
| Small Demersal Browser | *Chaetodon sedentarius* | 2010 - Pre-DWH | -1.13 | 0.32 | -3.55 | <0.01* |
|  |  | 2011 - Pre-DWH | -0.41 | 0.23 | -1.74 | 0.36 |
|  |  | 2012 - Pre-DWH | -0.68 | 0.24 | -2.83 | 0.03* |
|  |  | 2013 - Pre-DWH | -0.38 | 0.23 | -1.66 | 0.41 |
|  |  | 2014 - Pre-DWH | -0.75 | 0.26 | -2.82 | 0.03* |
|  |  | 2015 - Pre-DWH | -0.76 | 0.27 | -2.81 | 0.03* |
|  |  | 2016 - Pre-DWH | -0.77 | 0.29 | -2.66 | 0.05* |
|  |  | 2017 - Pre-DWH | -1.58 | 0.35 | -4.5 | <0.01* |
|  | *Parablennius marmoreus* | 2010 - Pre-DWH | -- | -- | -- | -- |
|  |  | 2011 - Pre-DWH | -0.27 | 0.35 | -0.79 | 0.94 |
|  |  | 2012 - Pre-DWH | -0.28 | 0.36 | -0.78 | 0.94 |
|  |  | 2013 - Pre-DWH | -0.41 | 0.41 | -1 | 0.84 |
|  |  | 2014 - Pre-DWH | -0.2 | 0.51 | -0.4 | 1 |
|  |  | 2015 - Pre-DWH | -- | -- | -- | -- |
|  |  | 2016 - Pre-DWH | 0.25 | 0.59 | 0.42 | 1 |
|  |  | 2017 - Pre-DWH | -0.71 | 0.6 | -1.2 | 0.71 |
|  | *Prognathodes aya* | 2010 - Pre-DWH | -1.43 | 0.44 | -3.25 | <0.01* |
|  |  | 2011 - Pre-DWH | -0.64 | 0.21 | -3.04 | 0.02* |
|  |  | 2012 - Pre-DWH | -0.8 | 0.22 | -3.62 | <0.01* |
|  |  | 2013 - Pre-DWH | -0.82 | 0.2 | -4.04 | <0.01* |
|  |  | 2014 - Pre-DWH | -0.61 | 0.24 | -2.47 | 0.08 |
|  |  | 2015 - Pre-DWH | -0.76 | 0.24 | -3.1 | 0.01* |
|  |  | 2016 - Pre-DWH | -- | -- | -- | -- |
|  |  | 2017 - Pre-DWH | -0.99 | 0.29 | -3.37 | <0.01* |
|  | *Stegastes leucostictus* | 2010 - Pre-DWH | -1.55 | 0.92 | -1.69 | 0.35 |
|  |  | 2011 - Pre-DWH | -0.83 | 0.55 | -1.5 | 0.47 |
|  |  | 2012 - Pre-DWH | -0.84 | 0.53 | -1.6 | 0.41 |
|  |  | 2013 - Pre-DWH | -0.91 | 0.47 | -1.91 | 0.23 |
|  |  | 2014 - Pre-DWH | -1.02 | 0.48 | -2.1 | 0.16 |
|  |  | 2015 - Pre-DWH | -1.33 | 0.51 | -2.6 | 0.05* |
|  |  | 2016 - Pre-DWH | -1.19 | 0.57 | -2.1 | 0.16 |
|  |  | 2017 - Pre-DWH | -1.42 | 0.56 | -2.55 | 0.06 |

**Table S6 Continued**

| Trophic Guild | Scientific Name | Comparison | Estimate | SE | *z* | *P* |
| --- | --- | --- | --- | --- | --- | --- |
| Small Demersal Browser | *Stegastes variabilis* | 2010 - Pre-DWH | -1.14 | 0.36 | -3.16 | <0.01* |
|  |  | 2011 - Pre-DWH | -1.12 | 0.35 | -3.17 | <0.01* |
|  |  | 2012 - Pre-DWH | -1.41 | 0.37 | -3.77 | <0.01* |
|  |  | 2013 - Pre-DWH | -1.23 | 0.31 | -3.93 | <0.01* |
|  |  | 2014 - Pre-DWH | -1.44 | 0.6 | -2.39 | 0.09 |
|  |  | 2015 - Pre-DWH | -- | -- | -- | -- |
|  |  | 2016 - Pre-DWH | -1.52 | 0.31 | -4.96 | <0.01* |
|  |  | 2017 - Pre-DWH | -- | -- | -- | -- |
| Large Demersal Browser | *Aluterus monoceros* | 2012 - 2011 | -0.02 | 0.5 | -0.04 | 1 |
|  |  | 2013 - 2011 | 0.67 | 0.48 | 1.4 | 0.48 |
|  |  | 2014 - 2011 | 0.22 | 0.47 | 0.47 | 0.99 |
|  |  | 2015 - 2011 | 0.16 | 0.64 | 0.26 | 1 |
|  |  | 2016 - 2011 | -- | -- | -- | -- |
|  |  | 2017 - 2011 | 0.16 | 0.64 | 0.26 | 1 |
|  | *Holacanthus bermudensis* | 2010 - Pre-DWH | -0.93 | 0.21 | -4.42 | <0.01* |
|  |  | 2011 - Pre-DWH | -0.47 | 0.17 | -2.79 | 0.03* |
|  |  | 2012 - Pre-DWH | -0.44 | 0.17 | -2.53 | 0.07 |
|  |  | 2013 - Pre-DWH | -0.33 | 0.16 | -2.01 | 0.22 |
|  |  | 2014 - Pre-DWH | -0.33 | 0.2 | -1.61 | 0.45 |
|  |  | 2015 - Pre-DWH | -0.52 | 0.21 | -2.44 | 0.08 |
|  |  | 2016 - Pre-DWH | -0.41 | 0.21 | -1.97 | 0.24 |
|  |  | 2017 - Pre-DWH | -0.56 | 0.23 | -2.38 | 0.1 |
|  | *Holacanthus ciliaris* | 2010 - Pre-DWH | 0.11 | 0.28 | 0.38 | 1 |
|  |  | 2011 - Pre-DWH | -0.8 | 0.19 | -4.16 | <0.01* |
|  |  | 2012 - Pre-DWH | -0.87 | 0.25 | -3.47 | <0.01* |
|  |  | 2013 - Pre-DWH | -0.59 | 0.21 | -2.76 | 0.04* |
|  |  | 2014 - Pre-DWH | -0.93 | 0.23 | -3.99 | <0.01* |
|  |  | 2015 - Pre-DWH | -1.16 | 0.3 | -3.94 | <0.01* |
|  |  | 2016 - Pre-DWH | -0.63 | 0.29 | -2.13 | 0.19 |
|  |  | 2017 - Pre-DWH | -1.04 | 0.3 | -3.45 | <0.01* |

**Table S6 Continued**

| Trophic Guild | Scientific Name | Comparison | Estimate | SE | *z* | *P* |
| --- | --- | --- | --- | --- | --- | --- |
| Small Demersal Invertivore | *Bodianus pulchellus* | 2010 - Pre-DWH | -0.77 | 0.29 | -2.64 | 0.05* |
|  |  | 2011 - Pre-DWH | -0.7 | 0.27 | -2.59 | 0.06 |
|  |  | 2012 - Pre-DWH | -0.32 | 0.29 | -1.11 | 0.79 |
|  |  | 2013 - Pre-DWH | -0.67 | 0.24 | -2.75 | 0.04* |
|  |  | 2014 - Pre-DWH | -0.86 | 0.31 | -2.72 | 0.04* |
|  |  | 2015 - Pre-DWH | -0.95 | 0.29 | -3.29 | <0.01* |
|  |  | 2016 - Pre-DWH | -1.24 | 0.3 | -4.16 | <0.01* |
|  |  | 2017 - Pre-DWH | -1.05 | 0.33 | -3.23 | <0.01* |
|  | Bodianus rufus | 2010 - Pre-DWH | -- | -- | -- | -- |
|  |  | 2011 - Pre-DWH | -0.48 | 0.41 | -1.17 | 0.64 |
|  |  | 2012 - Pre-DWH | -1.69 | 0.33 | -5.2 | <0.01* |
|  |  | 2013 - Pre-DWH | -0.54 | 0.32 | -1.73 | 0.29 |
|  |  | 2014 - Pre-DWH | -1.87 | 0.37 | -5 | <0.01* |
|  |  | 2015 - Pre-DWH | -1.69 | 0.42 | -4.07 | <0.01* |
|  |  | 2016 - Pre-DWH | -1.07 | 0.34 | -3.14 | <0.01* |
|  |  | 2017 - Pre-DWH | -1.96 | 0.41 | -4.76 | <0.01* |
|  | *Equetus lanceolatus* | 2010 - Pre-DWH | -1.52 | 0.47 | -3.2 | <0.01* |
|  |  | 2011 - Pre-DWH | -0.92 | 0.36 | -2.57 | 0.06 |
|  |  | 2012 - Pre-DWH | -0.69 | 0.4 | -1.7 | 0.4 |
|  |  | 2013 - Pre-DWH | -0.56 | 0.33 | -1.7 | 0.4 |
|  |  | 2014 - Pre-DWH | -1.15 | 0.37 | -3.09 | 0.01* |
|  |  | 2015 - Pre-DWH | -0.99 | 0.41 | -2.42 | 0.09 |
|  |  | 2016 - Pre-DWH | -0.62 | 0.43 | -1.43 | 0.59 |
|  |  | 2017 - Pre-DWH | -1.24 | 0.37 | -3.33 | <0.01* |
|  | *Haemulon aurolineatum* | 2010 - Pre-DWH | -0.62 | 0.7 | -0.88 | 0.94 |
|  |  | 2011 - Pre-DWH | -1.32 | 0.53 | -2.5 | 0.08 |
|  |  | 2012 - Pre-DWH | -0.1 | 0.51 | -0.2 | 1 |
|  |  | 2013 - Pre-DWH | -0.03 | 0.51 | -0.06 | 1 |
|  |  | 2014 - Pre-DWH | -0.93 | 0.57 | -1.64 | 0.44 |
|  |  | 2015 - Pre-DWH | -1.01 | 0.62 | -1.63 | 0.45 |
|  |  | 2016 - Pre-DWH | -0.67 | 0.62 | -1.08 | 0.84 |
|  |  | 2017 - Pre-DWH | -0.37 | 0.6 | -0.61 | 0.99 |

**Table S6 Continued**

| Trophic Guild | Scientific Name | Comparison | Estimate | SE | *z* | *P* |
| --- | --- | --- | --- | --- | --- | --- |
| Small Demersal Invertivore | *Halichoeres bivittatus* | 2010 - Pre-DWH | -1.63 | 0.91 | -1.79 | 0.39 |
|  |  | 2011 - Pre-DWH | -1.02 | 0.33 | -3.12 | 0.01* |
|  |  | 2012 - Pre-DWH | -0.7 | 0.34 | -2.07 | 0.23 |
|  |  | 2013 - Pre-DWH | -0.74 | 0.37 | -2.01 | 0.26 |
|  |  | 2014 - Pre-DWH | -1.22 | 0.46 | -2.65 | 0.06 |
|  |  | 2015 - Pre-DWH | -0.74 | 0.36 | -2.02 | 0.25 |
|  |  | 2016 - Pre-DWH | -1.58 | 0.41 | -3.83 | <0.01* |
|  |  | 2017 - Pre-DWH | -1.34 | 0.41 | -3.31 | <0.01* |
|  | *Holocentrus adscensionis* | 2010 - Pre-DWH | -- | -- | -- | -- |
|  |  | 2011 - Pre-DWH | -0.14 | 0.33 | -0.43 | 1 |
|  |  | 2012 - Pre-DWH | -0.17 | 0.29 | -0.59 | 0.99 |
|  |  | 2013 - Pre-DWH | -0.13 | 0.27 | -0.47 | 1 |
|  |  | 2014 - Pre-DWH | -0.58 | 0.48 | -1.22 | 0.71 |
|  |  | 2015 - Pre-DWH | -0.32 | 0.3 | -1.08 | 0.81 |
|  |  | 2016 - Pre-DWH | -0.5 | 0.48 | -1.05 | 0.83 |
|  |  | 2017 - Pre-DWH | -0.25 | 0.48 | -0.52 | 0.99 |
|  | *Holocentrus rufus* | 2012 - 2011 | 0.53 | 0.48 | 1.11 | 0.65 |
|  |  | 2013 - 2011 | -- | -- | -- | -- |
|  |  | 2014 - 2011 | 1.05 | 0.44 | 2.39 | 0.06 |
|  |  | 2015 - 2011 | -- | -- | -- | -- |
|  |  | 2016 - 2011 | 0.3 | 0.48 | 0.63 | 0.93 |
|  |  | 2017 - 2011 | -0.14 | 0.49 | -0.3 | 1 |
|  | *Pareques acuminatus* | 2010 - Pre-DWH | -- | -- | -- | -- |
|  |  | 2011 - Pre-DWH | 0.66 | 0.21 | 3.14 | <0.01* |
|  |  | 2012 - Pre-DWH | -0.64 | 0.22 | -2.89 | 0.02* |
|  |  | 2013 - Pre-DWH | 2.19 | 0.28 | 7.82 | <0.01* |
|  |  | 2014 - Pre-DWH | -0.96 | 0.23 | -4.12 | <0.01* |
|  |  | 2015 - Pre-DWH | -- | -- | -- | -- |
|  |  | 2016 - Pre-DWH | -1.02 | 0.22 | -4.64 | <0.01* |
|  |  | 2017 - Pre-DWH | -- | -- | -- | -- |

**Table S6 Continued**

| Trophic Guild | Scientific Name | Comparison | Estimate | SE | *z* | *P* |
| --- | --- | --- | --- | --- | --- | --- |
| Small Demersal Invertivore | *Pareques umbrosus* | 2010 - Pre-DWH | -1.56 | 0.48 | -3.27 | <0.01* |
|  |  | 2011 - Pre-DWH | -1.04 | 0.36 | -2.85 | 0.03* |
|  |  | 2012 - Pre-DWH | -0.83 | 0.38 | -2.19 | 0.16 |
|  |  | 2013 - Pre-DWH | -1.05 | 0.37 | -2.87 | 0.03* |
|  |  | 2014 - Pre-DWH | -0.89 | 0.39 | -2.26 | 0.14 |
|  |  | 2015 - Pre-DWH | 0.04 | 0.47 | 0.09 | 1 |
|  |  | 2016 - Pre-DWH | -1.65 | 0.47 | -3.53 | <0.01* |
|  |  | 2017 - Pre-DWH | -1.38 | 0.52 | -2.67 | 0.05* |
| Large Demersal Invertivore | *Archosargus probatocephalus* | 2010 - Pre-DWH | -- | -- | -- | -- |
|  |  | 2011 - Pre-DWH | -0.19 | 1.67 | -0.11 | 1 |
|  |  | 2012 - Pre-DWH | -0.12 | 1.16 | -0.1 | 1 |
|  |  | 2013 - Pre-DWH | -0.93 | 1.23 | -0.76 | 0.9 |
|  |  | 2014 - Pre-DWH | 0.45 | 1.27 | 0.36 | 1 |
|  |  | 2015 - Pre-DWH | -1.21 | 1.55 | -0.79 | 0.88 |
|  |  | 2016 - Pre-DWH | -- | -- | -- | -- |
|  |  | 2017 - Pre-DWH | -1.21 | 1.55 | -0.79 | 0.88 |
|  | *Balistes capriscus* | 2010 - Pre-DWH | -0.3 | 0.42 | -0.72 | 0.97 |
|  |  | 2011 - Pre-DWH | -0.54 | 0.32 | -1.68 | 0.39 |
|  |  | 2012 - Pre-DWH | -0.53 | 0.32 | -1.68 | 0.39 |
|  |  | 2013 - Pre-DWH | -0.49 | 0.3 | -1.61 | 0.44 |
|  |  | 2014 - Pre-DWH | -0.88 | 0.36 | -2.46 | 0.08 |
|  |  | 2015 - Pre-DWH | -0.25 | 0.37 | -0.67 | 0.98 |
|  |  | 2016 - Pre-DWH | -0.3 | 0.37 | -0.83 | 0.94 |
|  |  | 2017 - Pre-DWH | -0.17 | 0.36 | -0.48 | 1 |
|  | *Chilomycterus schoepfi* | 2012 - 2011 | 0.38 | 0.16 | 2.32 | 0.09 |
|  |  | 2013 - 2011 | -- | -- | -- | -- |
|  |  | 2014 - 2011 | 0.09 | 0.16 | 0.58 | 0.98 |
|  |  | 2015 - 2011 | -0.06 | 0.18 | -0.31 | 1 |
|  |  | 2016 - 2011 | 0.03 | 0.15 | 0.18 | 1 |
|  |  | 2017 - 2011 | -0.08 | 0.22 | -0.38 | 1 |

**Table S6 Continued**

| Trophic Guild | Scientific Name | Comparison | Estimate | SE | *z* | *P* |
| --- | --- | --- | --- | --- | --- | --- |
| Large Demersal Invertivore | *Pagrus pagrus* | 2010 - Pre-DWH | -1.76 | 0.48 | -3.7 | <0.01* |
|  |  | 2011 - Pre-DWH | -0.86 | 0.36 | -2.41 | 0.09 |
|  |  | 2012 - Pre-DWH | -1.16 | 0.4 | -2.86 | 0.03* |
|  |  | 2013 - Pre-DWH | -0.88 | 0.38 | -2.35 | 0.11 |
|  |  | 2014 - Pre-DWH | -0.98 | 0.44 | -2.23 | 0.15 |
|  |  | 2015 - Pre-DWH | -1.45 | 0.57 | -2.54 | 0.07 |
|  |  | 2016 - Pre-DWH | -1.69 | 0.54 | -3.15 | 0.01* |
|  |  | 2017 - Pre-DWH | -1.26 | 0.5 | -2.5 | 0.08 |
| Generalist Carnivore | *Caranx crysos* | 2010 - Pre-DWH | -1.18 | 1.42 | -0.83 | 0.92 |
|  |  | 2011 - Pre-DWH | -3.37 | 1.6 | -2.1 | 0.16 |
|  |  | 2012 - Pre-DWH | -0.67 | 1.3 | -0.52 | 0.99 |
|  |  | 2013 - Pre-DWH | -0.28 | 1.54 | -0.18 | 1 |
|  |  | 2014 - Pre-DWH | 2.43 | 1.42 | 1.71 | 0.34 |
|  |  | 2015 - Pre-DWH | -2.39 | 1.61 | -1.49 | 0.49 |
|  |  | 2016 - Pre-DWH | -- | -- | -- | -- |
|  |  | 2017 - Pre-DWH | -2.13 | 1.91 | -1.11 | 0.76 |
|  | *Centropristis ocyurus* | 2010 - Pre-DWH | -1.66 | 0.79 | -2.1 | 0.2 |
|  |  | 2011 - Pre-DWH | -0.42 | 0.31 | -1.35 | 0.68 |
|  |  | 2012 - Pre-DWH | -0.48 | 0.33 | -1.44 | 0.62 |
|  |  | 2013 - Pre-DWH | -0.46 | 0.32 | -1.44 | 0.61 |
|  |  | 2014 - Pre-DWH | -0.74 | 0.33 | -2.23 | 0.15 |
|  |  | 2015 - Pre-DWH | -0.92 | 0.38 | -2.46 | 0.09 |
|  |  | 2016 - Pre-DWH | -1.33 | 0.45 | -2.95 | 0.02* |
|  |  | 2017 - Pre-DWH | -1.56 | 0.59 | -2.65 | 0.05* |
|  | *Diplectrum formosum* | 2012 - 2011 | -0.25 | 0.64 | -0.4 | 0.97 |
|  |  | 2013 - 2011 | -0.4 | 0.63 | -0.64 | 0.87 |
|  |  | 2014 - 2011 | -0.61 | 0.84 | -0.73 | 0.82 |
|  |  | 2015 - 2011 | -0.35 | 0.62 | -0.56 | 0.91 |

**Table S6 Continued**

| Trophic Guild | Scientific Name | Comparison | Estimate | SE | *z* | *P* |
| --- | --- | --- | --- | --- | --- | --- |
| Generalist Carnivore | *Epinephelus cruentatus* | 2010 - Pre-DWH | -- | -- | -- | -- |
|  |  | 2011 - Pre-DWH | -1.06 | 0.33 | -3.17 | <0.01* |
|  |  | 2012 - Pre-DWH | -0.91 | 0.29 | -3.14 | <0.01* |
|  |  | 2013 - Pre-DWH | -1.07 | 0.27 | -4.03 | <0.01* |
|  |  | 2014 - Pre-DWH | -1.12 | 0.28 | -3.98 | <0.01* |
|  |  | 2015 - Pre-DWH | -0.93 | 0.3 | -3.15 | <0.01* |
|  |  | 2016 - Pre-DWH | -1.25 | 0.34 | -3.63 | <0.01* |
|  |  | 2017 - Pre-DWH | -0.92 | 0.3 | -3.11 | 0.01* |
|  | *Epinephelus morio* | 2010 - Pre-DWH | -0.77 | 0.13 | -5.92 | <0.01* |
|  |  | 2011 - Pre-DWH | -0.57 | 0.12 | -4.89 | <0.01* |
|  |  | 2012 - Pre-DWH | -0.6 | 0.12 | -4.85 | <0.01* |
|  |  | 2013 - Pre-DWH | -0.64 | 0.12 | -5.39 | <0.01* |
|  |  | 2014 - Pre-DWH | -0.8 | 0.17 | -4.8 | <0.01* |
|  |  | 2015 - Pre-DWH | -0.8 | 0.16 | -4.95 | <0.01* |
|  |  | 2016 - Pre-DWH | -0.75 | 0.25 | -2.98 | 0.02* |
|  |  | 2017 - Pre-DWH | -- | -- | -- | -- |
|  | *Liopropoma eukrines* | 2010 - Pre-DWH | -0.73 | 0.24 | -3.1 | 0.01* |
|  |  | 2011 - Pre-DWH | -0.21 | 0.21 | -1.04 | 0.8 |
|  |  | 2012 - Pre-DWH | -0.44 | 0.2 | -2.23 | 0.12 |
|  |  | 2013 - Pre-DWH | -0.38 | 0.19 | -1.94 | 0.22 |
|  |  | 2014 - Pre-DWH | -0.39 | 0.21 | -1.88 | 0.25 |
|  |  | 2015 - Pre-DWH | -0.47 | 0.21 | -2.22 | 0.12 |
|  |  | 2016 - Pre-DWH | -0.98 | 0.26 | -3.75 | <0.01* |
|  |  | 2017 - Pre-DWH | -0.14 | 0.26 | -0.54 | 0.99 |
|  | *Lutjanus campechanus* | 2010 - Pre-DWH | -1.07 | 0.38 | -2.86 | 0.03* |
|  |  | 2011 - Pre-DWH | -1.02 | 0.28 | -3.65 | <0.01* |
|  |  | 2012 - Pre-DWH | -1.13 | 0.29 | -3.86 | <0.01* |
|  |  | 2013 - Pre-DWH | -1.43 | 0.28 | -5.18 | <0.01* |
|  |  | 2014 - Pre-DWH | -1.64 | 0.32 | -5.15 | <0.01* |
|  |  | 2015 - Pre-DWH | -2.1 | 0.38 | -5.52 | <0.01* |
|  |  | 2016 - Pre-DWH | -2.06 | 0.37 | -5.62 | <0.01* |
|  |  | 2017 - Pre-DWH | -1.61 | 0.36 | -4.49 | <0.01* |

**Table S6 Continued**

| Trophic Guild | Scientific Name | Comparison | Estimate | SE | *z* | *P* |
| --- | --- | --- | --- | --- | --- | --- |
| Generalist Carnivore | *Lutjanus griseus* | 2010 - Pre-DWH | -1.28 | 0.56 | -2.27 | 0.14 |
|  |  | 2011 - Pre-DWH | -1.59 | 0.44 | -3.61 | <0.01* |
|  |  | 2012 - Pre-DWH | -1.02 | 0.44 | -2.34 | 0.12 |
|  |  | 2013 - Pre-DWH | -1 | 0.44 | -2.25 | 0.14 |
|  |  | 2014 - Pre-DWH | -2.07 | 0.52 | -3.97 | <0.01* |
|  |  | 2015 - Pre-DWH | -0.8 | 0.57 | -1.4 | 0.64 |
|  |  | 2016 - Pre-DWH | -2.31 | 0.6 | -3.84 | <0.01* |
|  |  | 2017 - Pre-DWH | -1.85 | 0.56 | -3.32 | <0.01* |
|  | *Lutjanus synagris* | 2010 - Pre-DWH | 0.74 | 0.83 | 0.89 | 0.96 |
|  |  | 2011 - Pre-DWH | -0.91 | 0.84 | -1.09 | 0.88 |
|  |  | 2012 - Pre-DWH | -1.13 | 0.52 | -2.19 | 0.18 |
|  |  | 2013 - Pre-DWH | 0.19 | 0.55 | 0.34 | 1 |
|  |  | 2014 - Pre-DWH | -1.58 | 0.64 | -2.48 | 0.09 |
|  |  | 2015 - Pre-DWH | -1.17 | 0.65 | -1.81 | 0.38 |
|  |  | 2016 - Pre-DWH | -1.17 | 0.92 | -1.28 | 0.77 |
|  |  | 2017 - Pre-DWH | -1.29 | 0.59 | -2.17 | 0.19 |
|  | *Pterois volitans* | 2012 - 2011 | 0.25 | 0.64 | 0.39 | 0.93 |
|  |  | 2013 - 2011 | 0.62 | 0.63 | 0.98 | 0.51 |
|  |  | 2014 - 2011 | 0.84 | 0.65 | 1.29 | 0.33 |
|  |  | 2015 - 2011 | 0.41 | 0.64 | 0.63 | 0.76 |
|  |  | 2016 - 2011 | 0.54 | 0.65 | 0.83 | 0.61 |
|  |  | 2017 - 2011 | 0.89 | 0.65 | 1.37 | 0.29 |
|  | *Rypticus maculatus* | 2010 - Pre-DWH | -0.53 | 0.44 | -1.22 | 0.71 |
|  |  | 2011 - Pre-DWH | -0.11 | 0.25 | -0.42 | 1 |
|  |  | 2012 - Pre-DWH | -0.2 | 0.27 | -0.76 | 0.96 |
|  |  | 2013 - Pre-DWH | -0.16 | 0.26 | -0.6 | 0.99 |
|  |  | 2014 - Pre-DWH | -0.27 | 0.29 | -0.94 | 0.89 |
|  |  | 2015 - Pre-DWH | -0.33 | 0.29 | -1.13 | 0.78 |
|  |  | 2016 - Pre-DWH | -0.22 | 0.44 | -0.51 | 1 |
|  |  | 2017 - Pre-DWH | -0.32 | 0.33 | -0.98 | 0.87 |

**Table S6 Continued**

| Trophic Guild | Scientific Name | Comparison | Estimate | SE | *z* | *P* |
| --- | --- | --- | --- | --- | --- | --- |
| Generalist Carnivore | *Seriola dumerili* | 2010 - Pre-DWH | 0.45 | 0.54 | 0.83 | 0.96 |
|  |  | 2011 - Pre-DWH | 0.76 | 0.45 | 1.69 | 0.43 |
|  |  | 2012 - Pre-DWH | -0.13 | 0.44 | -0.29 | 1 |
|  |  | 2013 - Pre-DWH | -0.58 | 0.44 | -1.33 | 0.69 |
|  |  | 2014 - Pre-DWH | -1.19 | 0.56 | -2.11 | 0.19 |
|  |  | 2015 - Pre-DWH | -0.96 | 0.51 | -1.88 | 0.31 |
|  |  | 2016 - Pre-DWH | -1.65 | 0.59 | -2.8 | 0.04* |
|  |  | 2017 - Pre-DWH | -1.26 | 0.56 | -2.27 | 0.14 |
|  | *Seriola rivoliana* | 2010 - Pre-DWH | -- | -- | -- | -- |
|  |  | 2011 - Pre-DWH | -0.87 | 0.37 | -2.36 | 0.11 |
|  |  | 2012 - Pre-DWH | -0.98 | 0.34 | -2.84 | 0.03* |
|  |  | 2013 - Pre-DWH | -0.57 | 0.32 | -1.8 | 0.35 |
|  |  | 2014 - Pre-DWH | -1.18 | 0.45 | -2.6 | 0.06 |
|  |  | 2015 - Pre-DWH | -0.45 | 0.36 | -1.22 | 0.77 |
|  |  | 2016 - Pre-DWH | -0.29 | 0.45 | -0.64 | 0.99 |
|  |  | 2017 - Pre-DWH | -1.33 | 0.45 | -2.94 | 0.02* |
|  | *Serranus phoebe* | 2010 - Pre-DWH | -0.91 | 0.56 | -1.65 | 0.39 |
|  |  | 2011 - Pre-DWH | -1.2 | 0.38 | -3.15 | 0.01* |
|  |  | 2012 - Pre-DWH | -0.26 | 0.39 | -0.68 | 0.97 |
|  |  | 2013 - Pre-DWH | -1.07 | 0.39 | -2.74 | 0.03* |
|  |  | 2014 - Pre-DWH | -1.1 | 0.4 | -2.78 | 0.03* |
|  |  | 2015 - Pre-DWH | -1.03 | 0.41 | -2.48 | 0.07 |
|  |  | 2016 - Pre-DWH | -1.36 | 0.47 | -2.91 | 0.02* |
|  |  | 2017 - Pre-DWH | -1.41 | 0.44 | -3.23 | <0.01* |
|  | *Serranus subligarius* | 2010 - Pre-DWH | -- | -- | -- | -- |
|  |  | 2011 - Pre-DWH | -0.3 | 0.35 | -0.88 | 0.95 |
|  |  | 2012 - Pre-DWH | 0.29 | 0.35 | 0.83 | 0.96 |
|  |  | 2013 - Pre-DWH | -0.4 | 0.46 | -0.88 | 0.95 |
|  |  | 2014 - Pre-DWH | 0.09 | 0.69 | 0.13 | 1 |
|  |  | 2015 - Pre-DWH | -1.16 | 0.52 | -2.21 | 0.16 |
|  |  | 2016 - Pre-DWH | -0.29 | 0.69 | -0.42 | 1 |
|  |  | 2017 - Pre-DWH | -0.29 | 0.69 | -0.42 | 1 |

**Table S6 Continued**

| Trophic Guild | Scientific Name | Comparison | Estimate | SE | *z* | *P* |
| --- | --- | --- | --- | --- | --- | --- |
| Piscivore | *Carcharhinus plumbeus* | 2010 - Pre-DWH | -- | -- | -- | -- |
|  |  | 2011 - Pre-DWH | 0.09 | 0.36 | 0.25 | 1 |
|  |  | 2012 - Pre-DWH | -0.27 | 0.25 | -1.09 | 0.85 |
|  |  | 2013 - Pre-DWH | -0.18 | 0.28 | -0.65 | 0.99 |
|  |  | 2014 - Pre-DWH | -0.99 | 0.36 | -2.73 | 0.04* |
|  |  | 2015 - Pre-DWH | -0.75 | 0.32 | -2.31 | 0.12 |
|  |  | 2016 - Pre-DWH | -0.75 | 0.24 | -3.07 | 0.01* |
|  |  | 2017 - Pre-DWH | -1.06 | 0.36 | -2.92 | 0.02* |
|  | *Mycteroperca microlepis* | 2010 - Pre-DWH | -1.11 | 0.39 | -2.82 | 0.03* |
|  |  | 2011 - Pre-DWH | -0.83 | 0.28 | -2.94 | 0.02* |
|  |  | 2012 - Pre-DWH | -0.83 | 0.25 | -3.34 | <0.01* |
|  |  | 2013 - Pre-DWH | -0.81 | 0.35 | -2.35 | 0.12 |
|  |  | 2014 - Pre-DWH | -1.03 | 0.26 | -4.02 | <0.01* |
|  |  | 2015 - Pre-DWH | -1.16 | 0.31 | -3.74 | <0.01* |
|  |  | 2016 - Pre-DWH | -0.61 | 0.34 | -1.79 | 0.39 |
|  |  | 2017 - Pre-DWH | -1.45 | 0.51 | -2.86 | 0.03* |
|  | *Mycteroperca phenax* | 2010 - Pre-DWH | -0.97 | 0.26 | -3.72 | <0.01* |
|  |  | 2011 - Pre-DWH | -0.47 | 0.19 | -2.44 | 0.09 |
|  |  | 2012 - Pre-DWH | -0.65 | 0.2 | -3.22 | <0.01* |
|  |  | 2013 - Pre-DWH | -0.67 | 0.2 | -3.38 | <0.01* |
|  |  | 2014 - Pre-DWH | -1.04 | 0.24 | -4.31 | <0.01* |
|  |  | 2015 - Pre-DWH | -0.7 | 0.25 | -2.74 | 0.04* |
|  |  | 2016 - Pre-DWH | -1.13 | 0.29 | -3.95 | <0.01* |
|  |  | 2017 - Pre-DWH | -1 | 0.26 | -3.83 | <0.01* |
| Reef Planktivore | *Apogon pseudomaculatus* | 2010 - Pre-DWH | -1.2 | 0.78 | -1.54 | 0.46 |
|  |  | 2011 - Pre-DWH | -0.41 | 0.52 | -0.8 | 0.94 |
|  |  | 2012 - Pre-DWH | -1.72 | 0.55 | -3.11 | 0.01* |
|  |  | 2013 - Pre-DWH | -0.02 | 0.56 | -0.04 | 1 |
|  |  | 2014 - Pre-DWH | -1.27 | 0.56 | -2.28 | 0.11 |
|  |  | 2015 - Pre-DWH | -0.96 | 0.58 | -1.66 | 0.38 |
|  |  | 2016 - Pre-DWH | 0.03 | 0.88 | 0.03 | 1 |
|  |  | 2017 - Pre-DWH | -- | -- | -- | -- |

**Table S6 Continued**

| Trophic Guild | Scientific Name | Comparison | Estimate | SE | *z* | *P* |
| --- | --- | --- | --- | --- | --- | --- |
| Reef Planktivore | *Baldwinella aureorubens* | 2012 - 2011 | -- | -- | -- | -- |
|  |  | 2013 - 2011 | 1.36 | 0.87 | 1.57 | 0.39 |
|  |  | 2014 - 2011 | 1.58 | 2.04 | 0.78 | 0.91 |
|  |  | 2015 - 2011 | 4.77 | 0.93 | 5.14 | <0.01* |
|  |  | 2016 - 2011 | 1.78 | 1.46 | 1.22 | 0.63 |
|  |  | 2017 - 2011 | 0.22 | 0.96 | 0.23 | 1 |
|  | *Chromis enchrysura* | 2010 - Pre-DWH | -0.32 | 0.79 | -0.4 | 1 |
|  |  | 2011 - Pre-DWH | -0.44 | 0.36 | -1.2 | 0.79 |
|  |  | 2012 - Pre-DWH | -0.65 | 0.39 | -1.67 | 0.44 |
|  |  | 2013 - Pre-DWH | 0.27 | 0.35 | 0.77 | 0.97 |
|  |  | 2014 - Pre-DWH | -0.46 | 0.4 | -1.16 | 0.81 |
|  |  | 2015 - Pre-DWH | 0.48 | 0.41 | 1.16 | 0.81 |
|  |  | 2016 - Pre-DWH | -0.67 | 0.45 | -1.49 | 0.57 |
|  |  | 2017 - Pre-DWH | -1.22 | 0.51 | -2.37 | 0.11 |
|  | *Paranthias furcifer* | 2010 - Pre-DWH | -1.68 | 0.76 | -2.23 | 0.13 |
|  |  | 2011 - Pre-DWH | 0.45 | 0.68 | 0.67 | 0.97 |
|  |  | 2012 - Pre-DWH | -0.27 | 0.64 | -0.42 | 1 |
|  |  | 2013 - Pre-DWH | -0.82 | 1.04 | -0.79 | 0.94 |
|  |  | 2014 - Pre-DWH | -1.2 | 0.68 | -1.75 | 0.34 |
|  |  | 2015 - Pre-DWH | 1.58 | 1.02 | 1.54 | 0.47 |
|  |  | 2016 - Pre-DWH | -- | -- | -- | -- |
|  |  | 2017 - Pre-DWH | -- | -- | -- | -- |
|  | *Priacanthus arenatus* | 2010 - Pre-DWH | -1.04 | 0.35 | -2.96 | 0.02* |
|  |  | 2011 - Pre-DWH | -0.37 | 0.26 | -1.42 | 0.6 |
|  |  | 2012 - Pre-DWH | -0.12 | 0.27 | -0.45 | 1 |
|  |  | 2013 - Pre-DWH | -0.29 | 0.25 | -1.14 | 0.8 |
|  |  | 2014 - Pre-DWH | -0.38 | 0.31 | -1.2 | 0.76 |
|  |  | 2015 - Pre-DWH | 0.67 | 0.8 | 0.83 | 0.95 |
|  |  | 2016 - Pre-DWH | -- | -- | -- | -- |
|  |  | 2017 - Pre-DWH | -0.04 | 0.49 | -0.08 | 1 |

**Table S6 Continued**

| Trophic Guild | Scientific Name | Comparison | Estimate | SE | *z* | *P* |
| --- | --- | --- | --- | --- | --- | --- |
| Reef Planktivore | *Pristigenys alta* | 2010 - Pre-DWH | -- | -- | -- | -- |
|  |  | 2011 - Pre-DWH | -0.21 | 0.59 | -0.36 | 1 |
|  |  | 2012 - Pre-DWH | -- | -- | -- | -- |
|  |  | 2013 - Pre-DWH | 0.47 | 0.59 | 0.79 | 0.92 |
|  |  | 2014 - Pre-DWH | -0.01 | 0.46 | -0.02 | 1 |
|  |  | 2015 - Pre-DWH | -0.26 | 0.37 | -0.7 | 0.95 |
|  |  | 2016 - Pre-DWH | 0.18 | 0.38 | 0.47 | 0.99 |
|  |  | 2017 - Pre-DWH | -0.02 | 0.39 | -0.06 | 1 |
|  | *Pronotogrammus martinicensis* | 2010 - Pre-DWH | 0.07 | 0.74 | 0.1 | 1 |
|  |  | 2011 - Pre-DWH | 0.58 | 0.57 | 1.01 | 0.87 |
|  |  | 2012 - Pre-DWH | 0.41 | 0.61 | 0.66 | 0.99 |
|  |  | 2013 - Pre-DWH | 0.56 | 0.55 | 1.01 | 0.87 |
|  |  | 2014 - Pre-DWH | 0.38 | 0.62 | 0.61 | 0.99 |
|  |  | 2015 - Pre-DWH | 0.67 | 0.65 | 1.04 | 0.86 |
|  |  | 2016 - Pre-DWH | 0.13 | 0.76 | 0.17 | 1 |
|  |  | 2017 - Pre-DWH | -0.58 | 0.77 | -0.76 | 0.97 |
|  | *Rhomboplites aurorubens* | 2010 - Pre-DWH | -0.53 | 0.6 | -0.88 | 0.94 |
|  |  | 2011 - Pre-DWH | -0.93 | 0.48 | -1.94 | 0.26 |
|  |  | 2012 - Pre-DWH | -1.52 | 0.48 | -3.15 | 0.01* |
|  |  | 2013 - Pre-DWH | -0.02 | 0.47 | -0.04 | 1 |
|  |  | 2014 - Pre-DWH | -0.72 | 0.55 | -1.3 | 0.69 |
|  |  | 2015 - Pre-DWH | 0.11 | 0.56 | 0.2 | 1 |
|  |  | 2016 - Pre-DWH | -0.34 | 0.56 | -0.61 | 0.99 |
|  |  | 2017 - Pre-DWH | 0.25 | 0.59 | 0.43 | 1 |
|  | *Stegastes partitus* | 2010 - Pre-DWH | -1.73 | 0.63 | -2.75 | 0.02* |
|  |  | 2011 - Pre-DWH | -- | -- | -- | -- |
|  |  | 2012 - Pre-DWH | -- | -- | -- | -- |
|  |  | 2013 - Pre-DWH | -1.01 | 0.86 | -1.18 | 0.58 |
|  |  | 2014 - Pre-DWH | -0.72 | 0.86 | -0.84 | 0.82 |
|  |  | 2015 - Pre-DWH | -0.9 | 0.93 | -0.97 | 0.72 |
|  |  | 2016 - Pre-DWH | -1.62 | 1.04 | -1.56 | 0.34 |
|  |  | 2017 - Pre-DWH | -- | -- | -- | -- |

**Table S6 Continued**

| Trophic Guild | Scientific Name | Comparison | Estimate | SE | *z* | *P* |
| --- | --- | --- | --- | --- | --- | --- |
| Reef Planktivore | Damselfish | 2010 - Pre-DWH | -3.08 | 1.18 | -2.6 | 0.06 |
|  |  | 2011 - Pre-DWH | -2.12 | 0.75 | -2.85 | 0.03* |
|  |  | 2012 - Pre-DWH | -0.58 | 0.92 | -0.63 | 0.99 |
|  |  | 2013 - Pre-DWH | 0.14 | 0.63 | 0.22 | 1 |
|  |  | 2014 - Pre-DWH | 0.9 | 0.76 | 1.18 | 0.79 |
|  |  | 2015 - Pre-DWH | 0.56 | 0.7 | 0.8 | 0.96 |
|  |  | 2016 - Pre-DWH | -0.43 | 1.01 | -0.42 | 1 |
|  |  | 2017 - Pre-DWH | 0.36 | 0.78 | 0.46 | 1 |

**Figure S1.** Standardized trophic guild density (±95% CIs) estimates for small demersal invertivores with (A) and without (B) tomtate. An asterisk (*) denotes a significant difference for the log-normal model (Table S4).

**Figure S1.**


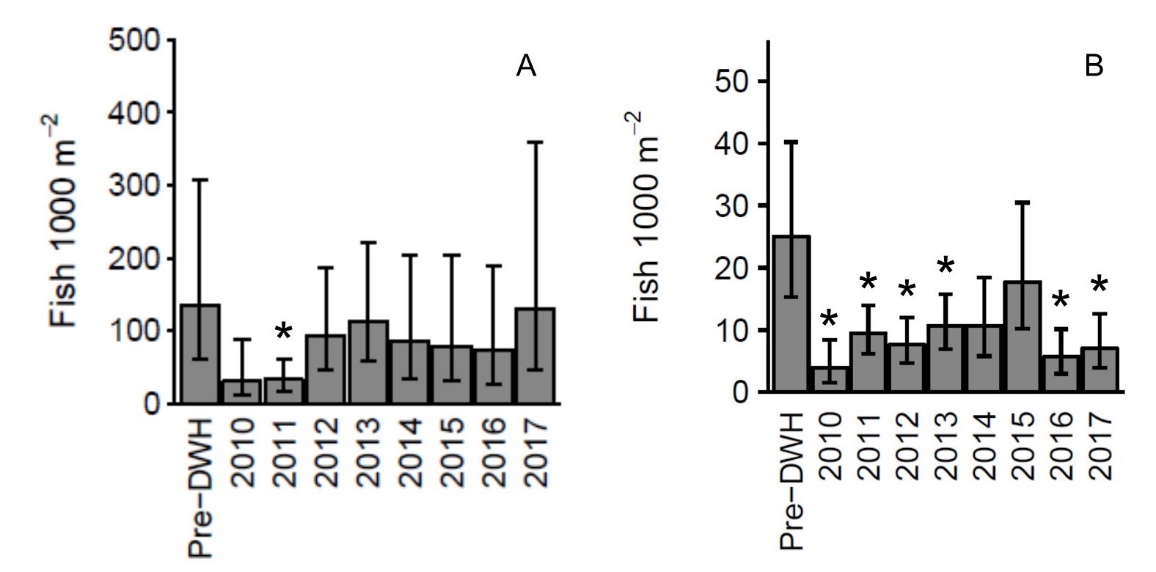

Supplement: Supplementary file 1 — Supplemental Materials. [file 41598_2020_62574_MOESM1_ESM.docx]
