# Supplementary material for: Changes in Reef Fish Community Structure Following the Deepwater Horizon Oil Spill
Source: Sci Rep. 2020 Apr 9;10:5621. doi: 10.1038/s41598-020-62574-y (PMC7145834; doi:10.1038/s41598-020-62574-y)
Supplement: Supplementary file 2 — Appendix A. [file 41598_2020_62574_MOESM2_ESM.docx]

*The following appendix accompanies the article*

**Changes in Reef Fish Community Structure Following the Deepwater Horizon Oil Spill**

**Justin P. Lewis^*^, Joseph H. Tarnecki, Steven B. Garner, David D. Chagaris, and William F. Patterson III**

^*^corresponding author: [justin.lewis@ufl.edu](mailto:justin.lewis@ufl.edu)

Appendix A.

| Trophic Guild | Family | Scientific Name | Common Name | Reference |
| --- | --- | --- | --- | --- |
| Herbivore | Acanthuridae | *Acanthurus chirurgus* | Doctorfish | ^1,2^ |
|  | Pomacanthidae | *Centropyge argi* | Cherubfish | ^2^ |
|  | Pomacentridae | *Microspathodon chrysurus* | Yellowtail Damselfish | ^3^ |
| Small Demersal Browser | Blenniidae | *Parablennius marmoreus* | Seaweed Blenny | ^2^ |
|  | Chaetodontidae | *Chaetodon ocellatus* | Spotfin Butterflyfish | ^4,5^ |
|  | Chaetodontidae | *Chaetodon sedentarius* | Reef Butterflyfish | ^2,6^ |
|  | Chaetodontidae | *Prognathodes aya* | Bank Butterflyfish | ^6^ |
|  | Monocanthidae | *Stephanolepis hispidus* | Planehead Filefish |  |
|  | Monocanthidae | *Stephanolepis setifer* | Pygmy Filefish | ^7^ |
|  | Pomacentridae | *Stegastes leucostictus* | Beaugregory | ^2,3^ |
|  | Pomacentridae | *Stegastes variabilis* | Cocoa Damselfish | ^3,8^ |
|  | Tetraodontidae | *Canthigaster rostrata* | Sharpnose Puffer | ^2^ |
|  | Tetraodontidae | *Sphoeroides parvus* | Least Puffer |  |
| Large Demersal Browser | Ephippidae | *Chaetodipterus faber* | Spadefish | ^2,9^ |
|  | Monocanthidae | *Aluterus monoceros* | Unicorn Filefish |  |
|  | Monocanthidae | *Aluterus schoepfii* | Orange Filefish | ^2,10^ |
|  | Monocanthidae | *Aluterus scriptus* | Scrawled Filefish | ^2^ |
|  | Pomacanthidae | *Holacanthus bermudensis* | Blue Angelfish | ^2^ |
|  | Pomacanthidae | *Holacanthus ciliaris* | Queen Angelfish | ^2^ |
|  | Pomacanthidae | *Holacanthus tricolor* | Rock Beauty Angelfish | ^2,11^ |
|  | Pomacanthidae | *Pomacanthus arcuatus* | Gray Angelfish | ^2,11^ |
|  | Pomacanthidae | *Pomacanthus paru* | French Angelfish | ^2,11^ |
| Small Demersal Invertivore | Haemulidae | *Anisotremus virginicus* | Porkfish | ^2^ |
|  | Haemulidae | *Haemulon aurolineatum* | Tomtate | ^2,12^ |
|  | Holocentridae | *Corniger spinosus* | Spinycheek Soldierfish | ^6^ |
|  | Holocentridae | *Holocentrus adscensionis* | Squirrelfish | ^2^ |
|  | Holocentridae | *Holocentrus rufus* | Longspine Squirrelfish | ^2^ |
|  | Holocentridae | *Plectrypops retrospinis* | Cardinal Soilderfish | ^13^ |
|  | Labridae | *Bodianus pulchellus* | Spotfin Hogfish |  |
|  | Labridae | *Bodianus rufus* | Spanish Hogfish | ^2^ |
|  | Labridae | *Halichoeres bathyphilus* | Greenband Wrasse |  |
|  | Labridae | *Halichoeres bivittatus* | Slippery Dick | ^2,14^ |
|  | Labridae | *Thalassoma bifasciatum* | Bluehead Wrasse | ^14^ |
|  | Labridae | *Xyrichtys novacula* | Pearly Razorfish |  |
|  | Microdesmidae | *Ptereleotris calliura* | Blue Goby |  |
|  | Sciaenidae | *Equetus lanceolatus* | Jacknife fish | ^2,15,16^ |
|  | Sciaenidae | *Equetus punctatus* | Spotted Drum | ^2^ |
|  | Sciaenidae | *Micropogonias undulatus* | Atlantic Croaker | ^17^ |
|  | Sciaenidae | *Pareques acuminatus* | Highhat | ^2^ |
|  | Sciaenidae | *Pareques iwamotoi* | Blackbar Drum | ^6^ |
|  | Sciaenidae | *Pareques umbrosus* | Cubbyu | ^6,16^ |
|  | Triglidae | *Bellator militaris* | Horned Searobin |  |

Appendix A continued.

| Trophic Guild | Family | Scientific Name | Common Name | Reference |
| --- | --- | --- | --- | --- |
| Large Demersal Invertivore | Balistidae | *Balistes capriscus* | Gray Triggerfish | ^18^ |
|  | Carangidae | *Alectis ciliaris* | African Pompano | ^19^ |
|  | Dasyatidae | *Dasyatis americana* | Southern Stingray | ^20^ |
|  | Dasyatidae | *Dasyatis centroura* | Roughtail Stingray | ^21^ |
|  | Dasyatidae | *Dasyatis sabina* | Atlantic Stingray | ^22^ |
|  | Dasyatidae | *Urobatis jamaicensis* | Yellow Stingray | ^23^ |
|  | Diodontidae | *Chilomycterus schoepfi* | Striped Burrfish | ^24^ |
|  | Ostraciidae | *Acanthostracion quadricornis* | Scrawled Cowfish | ^2^ |
|  | Rajidae | *Raja ackleyi* | Ocellate Skate |  |
|  | Sciaenidae | *Pogonias cromis* | Black Drum | ^25^ |
|  | Serranidae | *Alphestes afer* | Mutton Hamlet | ^2^ |
|  | Sparidae | *Archosargus probatocephalus* | Sheepshead | ^2,26^ |
|  | Sparidae | *Calamus bajonado* | Jolthead Porgy | ^2^ |
|  | Sparidae | *Calamus calamus* | Saucereye Porgy | ^2^ |
|  | Sparidae | *Calamus leucosteus* | Whitebone Porgy | ^27^ |
|  | Sparidae | *Calamus nodosus* | Knobbed Porgy | ^28^ |
|  | Sparidae | *Calamus proridens* | Littlehead Porgy | ^29^ |
|  | Sparidae | *Pagrus pagrus* | Red Porgy | ^6^ |
|  | Sparidae | *Stenotomus caprinus* | Longspine Porgy | ^27^ |
|  | Squalidae | *Mustelus canis* | Smooth Dogfish | ^30^ |
|  | Squalidae | *Mustelus norrisi* | Florida Smoothhound |  |
| Generalist Carnivore | Batrachoididae | *Opsanus beta* | Gulf toadfish | ^31^ |
|  | Batrachoididae | *Opsanus pardus* | Leopard Toadfish | ^6^ |
|  | Carangidae | *Caranx crysos* | Blue Runner | ^2,32,33^ |
|  | Carangidae | *Elagatis bipinnulata* | Rainbow runner | ^34^ |
|  | Carangidae | *Seriola dumerili* | Greater Amberjack | ^2,19,27,35^ |
|  | Carangidae | *Seriola rivoliana* | Almaco Jack | ^35^ |
|  | Echeneidae | *Echeneis naucrates* | Sharksucker | ^2^ |
|  | Echeneidae | *Remora remora* | Remora | ^2^ |
|  | Haemulidae | *Orthopristis chrysoptera* | Pigfish | ^36^ |
|  | Lutjanidae | *Lutjanus campechanus* | Red Snapper | ^37,38^ |
|  | Lutjanidae | *Lutjanus griseus* | Gray Snapper | ^39^ |
|  | Lutjanidae | *Lutjanus synagris* | Lane Snapper | ^2,39^ |
|  | Malacanthidae | *Malacanthus plumieri* | Sand Tilefish | ^2,6^ |
|  | Muraenidae | *Echidna catenata* | Chain Moray Eel | ^2,40^ |
|  | Muraenidae | *Gymnothorax funebris* | Green Moray Eel | ^19^ |
|  | Muraenidae | *Gymnothorax moringa* | Spotted Moray Eel | ^2,41,42^ |
|  | Ogcocephalidae | *Ogcocephalus corniger* | Longnose Batfish |  |
|  | Paralichthyidae | *Paralichthys albigutta* | Gulf Flounder | ^43^ |
|  | Rachycentridae | *Rachycentron canadum* | Cobia | ^44^ |
|  | Sciaenidae | *Sciaenops ocellatus* | Red Drum | ^45,46^ |
|  | Scorpaenidae | *Pterois volitans* | Red Lionfish | ^47,48^ |
|  | Scorpaenidae | *Scorpaena brasiliensis* | Barbfish | ^2^ |
|  | Scorpaenidae | *Scorpaena plumieri* | Spotted Scorpionfish | ^2^ |
|  | Serranidae | *Centropristis ocyurus* | Bank Sea Bass | ^6,49^ |
|  | Serranidae | *Diplectrum formosum* | Sand Perch | ^50,51^ |

Appendix A continued.

| Trophic Guild | Family | Scientific Name | Common Name | Reference |
| --- | --- | --- | --- | --- |
| Generalist Carnivore | Serranidae | *Epinephelus adscensionis* | Rock Hind | ^2,49^ |
|  | Serranidae | *Epinephelus cruentatus* | Graysby | ^2^ |
|  | Serranidae | *Epinephelus guttatus* | Red Hind | ^2,52,53^ |
|  | Serranidae | *Epinephelus morio* | Red Grouper | ^2,49^ |
|  | Serranidae | *Gonioplectrus hispanus* | Spanish Flag |  |
|  | Serranidae | *Hypoplectrus indigo* | Indigo Hamlet | ^49^ |
|  | Serranidae | *Hyporthodus niveatus* | Snowy Grouper | ^54^ |
|  | Serranidae | *Liopropoma eukrines* | Wrasse Bass | ^6^ |
|  | Serranidae | *Rypticus maculatus* | Whitespotted Soapfish | ^49^ |
|  | Serranidae | *Serranus phoebe* | Tattler | ^49^ |
|  | Serranidae | *Serranus subligarius* | Belted Sandfish | ^49,55^ |
| Piscivore | Antennariidae | *Fowlericthys ocellatus* | Ocellated Frogfish |  |
|  | Belonidae | *Tylosurus crocodilus* | Houndfish | ^2^ |
|  | Carangidae | *Seriola fasciata* | Lesser Amberjack |  |
|  | Carangidae | *Seriola zonata* | Banded Rudderfish |  |
|  | Carcharhinidae | *Carcharhinus falciformis* | Silky Shark | ^56^ |
|  | Carcharhinidae | *Carcharhinus leucas* | Bull Shark | ^57^ |
|  | Carcharhinidae | *Carcharhinus limbatus* | Blacktip | ^58^ |
|  | Carcharhinidae | *Carcharhinus obscurus* | Dusky Shark | ^30^ |
|  | Carcharhinidae | *Carcharhinus plumbeus* | Sandbar shark | ^59^ |
|  | Carcharhinidae | *Rhizoprionodon terraenovae* | Atlantic Sharpnose Shark | ^60–62^ |
|  | Fistulariidae | *Fistularia petimba* | Red Cornetfish |  |
|  | Muraenidae | *Muraena retifera* | Reticulate Moray | ^63^ |
|  | Paralichthyidae | *Paralichthys lethostigma* | Southern Flounder | ^64^ |
|  | Pomatomidae | *Pomatomus saltatrix* | Bluefish | ^65^ |
|  | Scombridae | *Scomberomorus cavalla* | King Mackerel | ^2^ |
|  | Scombridae | *Scomberomorus maculatus* | Spanish Mackerel |  |
|  | Serranidae | *Mycteroperca microlepis* | Gag | ^49,66^ |
|  | Serranidae | *Mycteroperca phenax* | Scamp | ^67^ |
|  | Sphyraenidae | *Sphyraena barracuda* | Great Barracuda | ^2,68^ |
|  | Synodontidae | *Synodus intermedius* | Sand Diver | ^2^ |
| Reef Planktivore | Apogonidae | *Apogon pseudomaculatus* | Twospot Cardinalfish | ^6^ |
|  | Apogonidae | *Paroncheilus affinis* | Bigtooth Cardinalfish |  |
|  | Holocentridae | *Myripristis jacobus* | Blackbar Soldierfish | ^2,19^ |
|  | Lutjanidae | *Rhomboplites aurorubens* | Vermilion Snapper | ^69^ |
|  | Pomacentridae | *Chromis cyanea* | Blue Chromis | ^2,3^ |
|  | Pomacentridae | *Chromis enchrysura* | Yellowtail Reeffish | ^3^ |
|  | Pomacentridae | *Stegastes partitus* | Bicolor Damselfish | ^3^ |
|  | Pomacentridae | Damselfish | Purple reeffish/dusky damselfish | ^2,3,70^ |

Appendix A continued.

| Trophic Guild | Family | Scientific Name | Common Name | Reference |
| --- | --- | --- | --- | --- |
| Reef Planktivore | Priacanthidae | *Priacanthus arenatus* | Atlantic Bigeye | ^2,6,27,71^ |
|  | Priacanthidae | *Pristigenys alta* | Short Bigeye | ^6^ |
|  | Serranidae | *Baldwinella aureorubens* | Streamer Bass |  |
|  | Serranidae | *Baldwinella vivanus* | Red Barbier | ^49,72^ |
|  | Serranidae | *Hemanthias leptus* | Longtail Bass | ^49^ |
|  | Serranidae | *Paranthias furcifer* | Creolefish | ^2,49,73^ |
|  | Serranidae | *Pronotogrammus martinicensis* | Roughtongue Bass | ^49^ |
| Pelagic Planktivore | Carangidae | *Decapterus macarellus* | Mackerel Scad | ^2^ |
|  | Carangidae | *Selar crumenophthalmus* | Bigeye Scad | ^2^ |
|  | Carangidae | *Selene setapinnis* | Atlantic Moonfish |  |
|  | Myliobatidae | *Manta birostris* | Manta Ray |  |

**References**

1. Dias, T. L. P., Rosa, I. L. & Feitoza, B. M. Food resource and habitat sharing by the three western South Atlantic surgeonfishes (Teleostei: Acanthuridae: Acanthurus) off Paraíba coast, north-eastern Brazil. *aqua, Int. J. Ichthyol.* **5**, 1–10 (2001).

2. Randall, J. E. Food habits of reef fishes of the West Indies. *Hawaii Inst. Mar. Biol.* 665–847 (1967).

3. Emery, A. R. Comparative ecology and functional osteology of fourteen species of damselfish (Pisces: Pomacentridae) at Alligator Reef, Florida Keys. *Bull. Mar. Sci.* **23**, 649–770 (1973).

4. Motta, P. J. Dentition patterns among Pacific and Western Atlantic butterflyfishes (Perciformes, Chaetodontidae): relationship to feeding ecology and evolutionary history. *Environ. Biol. Fishes* **25**, 159–170 (1989).

5. Aiken, K. *The Biology, Ecology, and Bionomics of Butterfly and Angelfishes, Chaetodontidae*. *Carribean Coral Reef Fishery Ressources* (International Center for Living Aquatic Resources Management, 1983).

6. Weaver, D. C., Dennis, G. D. & Sulak, K. J. *Community Structure and Trophic Ecology of Fishes on the Pinnacles Reef Tract*. *Final Synthesis Report* (2002).

7. Clements, W. H. & Livingston, R. J. Overlap and pollution-pnduced variability in the feeding habits of filefish (Pisces: Monacanthidae) from Apalachee Bay, Florida. *Copeia* **1983**, 331 (1983).

8. Nelson, B. D. & Bortone, S. A. Feeding guilds among artificial-reef fishes Northern Gulf of Mexico. *Gulf Mex. Sci.* **2**, 66–80 (1996).

9. Hayse, J. W. Feeding habits, age, growth, and reproduction of Atlantic spadefish *Chaetodipterus faber* (Pisces: Ephippidae) in South Carolina. *Fish. Bull.* **88**, 67–83 (1990).

10. Cargo, D. G. & Schultz, L. P. Notes on the biology of the sea nettle, *Chrysaora quinquecirrha*, in Chesapeake Bay. *Chesap. Sci.* **7**, 95 (1966).

11. Hourigan, T. F., Stanton, F. G., Motta, P. J., Kelley, C. D. & Carlson, B. The feeding ecology of three species of Caribbean angelfishes (family Pomacanthidae). *Environ. Biol. Fishes* **24**, 105–116 (1989).

12. Norberg, M. J. The ecology of tomtate, *Haemulon aurolineatum*, in the northern Gulf of Mexico and effects of the Deepwater Horizon oil spill. (University of South Alabama, 2015).

13. Gladfelter, W. B. & Johnson, W. S. Feeding niche separation in a guild of tropical reef fishes (Holocentridae). *Ecology* **64**, 552–563 (1983).

14. Clifton, K. B. & Motta, P. J. Feeding morphology, diet, and ecomorphological relationships among five Caribbean labrids (Teleostei, Labridae). *Copeia* **1998**, 953 (1998).

15. Lowe, R. H. The sciaenid fishes of Bristh Guiana. *Bull. Mar. Sci.* **16**, 26–57 (1966).

16. Darovev, J. E. *Sciaenid fishes (Osteichthyes: Perciformes) of western peninsular Florida*. *Memoirs of the Hourglass Cruises* **6**, (1983).

17. Overstreet, R. M. & Heard, R. W. Food of the Atlantic croaker, *Micropogonias undulatus*, from Mississippi Sound and the Gulf of Mexico. *Gulf Res. Reports* **6**, (1978).

18. Vose, F. E. & Nelson, W. G. Gray triggerfish (*Balistes capriscus* Gmelin) feeding from artifical and natural substrate in shallow Atlantic waters of Florida. *Bull. Mar. Sci.* **55**, 1316–1323 (1994).

19. Bohnsack, J. A., Harper, D. E., McClellan, D. B., Sutherland, D. L. & White, M. W. Resource survey of fishes within Looe Key National Marine Sanctuary. *NOAA Tech. Memo.* 1–108 (1987).

20. Gilliam, D. & Sullivan, K. M. Diet and feeding habits of the southern stingray *Dasyatis americana* in the central Bahamas. *Bull. Mar. Sci.* **52**, 1007–1013 (1993).

21. Struhsaker, P. Observations on the biology and distribution of the thorny stingray, *Dasyatis centroura* (Pisces: Dasyatidae). *Bull. Mar. Sci.* **19**, 456–481 (1969).

22. Snelson, F. F. & Williams, S. E. Notes on the occurrence, distribution, and biology of elasmobranch fishes in the Indian River Lagoon system, Florida. *Estuaries* **4**, 110 (1981).

23. O’Shea, O. R., Wueringer, B. E., Winchester, M. M. & Brooks, E. J. Comparative feeding ecology of the yellow ray urobatis jamaicensis (Urotrygonidae) from the bahamas. *J. Fish Biol.* **92**, 73–84 (2018).

24. Motta, P. J. *et al.* Feeding relationships among nine species of seagrass fishes of Tampa Bay, Florida. *Bull. Mar. Sci.* **56**, 185–200 (1995).

25. Overstreet, R. M. & Heard, R. W. Food contents of six commercial fishes from Mississippi Sound. *Gulf Res. Reports* **7**, (1982).

26. Sedberry, G. R. Feeding habits of sheepshead, *Archosargus probatocephalus*, in offshore reef habitats of the southeastern continental shelf. *Northeast Gulf Sci.* **9**, (1987).

27. Bowman, R. E., Stillwell, C. E., Michaels, W. L. & Grosslein, M. D. National Oceanic and Atmospheric Administration National Marine Fisheries Service Food of Northwest Atlantic Fishes and Two Common Species of Squid. *NOAA Tech. Memo. NMFS-NE-155* (2000).

28. Horvath, M. L., Grimes, C. B., Huntsman, G. R. & Carolina, S. Growth, mortality, reproduction and feeding of knobbed porgy, *Calamus nodosus*, along the southeastern United States coast. *Bull. Mar. Sci.* **46**, 677–687 (1990).

29. Darcy, G. H. *Synopsis of biological data on the porgies, Calamus arctifrons and C. proridens (Pisces: Sparidae)*. (1986).

30. Gelsleichter, J., Musick, J. A. & Nichols, S. Food habits of the smooth dogfish, *Mustelus canis*, dusky shark, *Carcharhinus obscurus*, Atlantic sharpnose shark, *Rhizoprionodon terraenovae*, and the sand tiger, *Carcharias taurus*, from the northwest Atlantic Ocean. *Environ. Biol. Fishes* **54**, 205–217 (1999).

31. Springer, V. G. & Woodburn, K. D. *An ecological study of the fishes of the Tampa Bay area*. *Florida State Board of Conservation* (1960).

32. Keenan, S. F. The importance of zooplankton in the diets of the blue runner (*Caranx crysos*) near offshore petroleum platforms in the northern Gulf of Mexico. 166 (1996).

33. Sley, A., Jarboui, O., Ghorbel, M. & Bouain, A. Food and feeding habits of *Caranx crysos* from the Gulf of Gabs (Tunisia). *J. Mar. Biol. Assoc. United Kingdom* **89**, 1375–1380 (2009).

34. Ménard, F. *et al.* Pelagic cephalopods in the western Indian Ocean: New information from diets of top predators. *Deep. Res. Part II Top. Stud. Oceanogr.* **95**, 83–92 (2013).

35. Manooch, C. S. I. & Haimovici, M. Foods of greater amberjack, *Seriola dumerili*, and Almaco Jack, *Seriola rivoliana* (Pisces: Carangidae), from the South Atlantic Bight. *The Journal of the Elisha Mitchell Scientific Society* **99**, 1–9 (1983).

36. Howe, J. C. Diet Composition of Juvenile Pigfish, Orthopristis chrysoptera (Perciformes: Haemulidae), from the Northern Gulf of Mexico. *Gulf Mex. Sci.* **19**, 55–60 (2001).

37. Wells, R. J. D., Cowan, J. H. & Fry, B. Feeding ecology of red snapper *Lutjanus campechanus* in the northern Gulf of Mexico. *Mar. Ecol. Prog. Ser.* **361**, 213–225 (2008).

38. Tarnecki, J. H. & Patterson, W. F. Changes in red snapper diet and trophic ecology following the Deepwater Horizon oil spill. *Mar. Coast. Fish.* **7**, 135–147 (2015).

39. Franks, J. S. & VanderKooy, K. E. Feeding habits of juvenile lane snapper *Lutjanus synagris* from Mississippi coastal waters, with comments on the diet of gray snapper *Lutjanus griseus*. *Gulf Caribb. Res.* **12**, 11–17 (2000).

40. Mehta, R. S. Ecomorphology of the moray bite: relationship between dietary extremes and morphological diversity. *Physiol. Biochem. Zool.* **82**, 90–103 (2009).

41. Zokan, M. A. The life history of morays (Anguilliformes: Muraenidae) off the southeastern Atlantic coast of the United States. *Masters Abstr. Int. Vol. 46, no. 05, 118 p. 2008.* 1–118 (2008).

42. Young, R. F. & Winn, H. E. Activity patterns , diet , and shelter site use for two species of moray eels , *Gymnothorax moringa* and *Gymnothorax vicinus*, in Belize. *Am. Soc. Ichthyol. Herpetol.* **2003**, 44–55 (2003).

43. Peebles, E. B. & Hopkins, T. L. *Feeding habits of eight fish species from Tampa Bay, with observations on opportunistic predation*. (1993).

44. Meyer, G. H. & Franks, J. S. Food of Cobia, *Rachycentron canadum*, from the northcentral Gulf of Mexico. *Gulf Res. Reports* **9**, 161–167 (1996).

45. Scharf, F. S. & Schlight, K. K. Feeding habits of red drum (*Sciaenops ocellatus*) in Galveston Bay, Texas: seasonal diet variation and predator-prey size relationships. *Estuaries* **23**, 128 (2000).

46. Overstreet, R. M. & Heard, R. W. Food of the red drum, *Sciaenops ocellata*, from Mississippi Sound. *Gulf Res. Reports* **6**, 131–135 (1978).

47. Dahl, K. A., Patterson III, W. F., Robertson, A. & Ortmann, A. C. DNA barcoding significantly improves resolution of invasive lionfish diet in the northern Gulf of Mexico. *Biol. Invasions* **19**, 1917–1933 (2017).

48. Dahl, K. A. & Patterson III, W. F. Habitat-specific density and diet of rapidly expanding invasive red lionfish, *Pterois volitans*, populations in the northern Gulf of Mexico. *PLoS One* **9**, e105852 (2014).

49. Bullock, L. H. & Smith, G. B. *Seabasses (Pisces: Serranidae)*. (1991).

50. Bortone. *Studies on the biology of the sand perch Diplectrum formosum, Serranidae*. (1971).

51. Darcy, G. H. *Synopsis of Biological Data on the Sand Perch, Diplectrum formosum (Pisces: Serranidae)*. (1985).

52. Menzel, D. W. Utilization of food by a Bermuda reef fish, *Epinephelus guttatus*. *ICES J. Mar. Sci.* **25**, 216–222 (1960).

53. Thompson, R. & Munro, J. L. Aspects of the biology and ecology of Caribbean reef fishes: Serranidae (hinds and groupers). *J. Fish Biol.* **12**, 115–146 (1978).

54. Bielsa, L. M. & Labisky, R. F. Food habits of blueline tilefish, *Caulolatilus microps*, and snowy grouper, *Epinephelus niveatus*, from the lower Florida Keys. *Northeast Gulf Sci.* **9**, (1987).

55. Hastings, P. A. & Bortone, S. A. Observations on the life history of the belted sandfish, *Serranus subligarius* (Serranidae). *Environ. Biol. Fishes* **5**, 365–374 (1980).

56. Bonfil, R. S. The biology and ecology of the silky shark, *Carcharhinus falciformis*. in *Sharks of the open ocean: biology, fisheries and conservation* (eds. Camhi, M. D., Pikitch, E. K. & Babcock, E. A.) 114–127 (Blackwell Publishing, 2008).

57. Snelson, F. F., Mulligan, T. J. & Williams, S. E. Food habits, occurrence, and population structure of the bull shark, *Carcharhinus leucas*, in Florida coastal lagoons. *Bull. Mar. Sci.* **34**, 71–80 (1984).

58. Castro, J. Biology of the blacktip shark, Carcharhinus limbatus, off the southeastern United States. *Bull. Mar. Sci.* **59**, 508–522 (1996).

59. Stillwell, C. E. & Kohler, N. E. Food habits of the sandbar shark *Carcharhinus plumbeus* off the U.S. northeast coast, with estimates of daily ration. *Fish. Bullitin* **91**, 138–150 (1992).

60. Bethea, D. M., Carlson, J. K., Buckel, J. A. & Satterwhite, M. Ontogenetic and site-related trends in the diet of the Atlantic sharpnose shark *Rhizoprionodon terraenovae* from the northeast Gulf of Mexico. *Bull. Mar. Sci.* **78**, 287–307 (2006).

61. Drymon, J. M., Powers, S. P. & Carmichael, R. H. Trophic plasticity in the Atlantic sharpnose shark (*Rhizoprionodon terraenovae*) from the north central Gulf of Mexico. *Environ. Biol. Fishes* **95**, 21–35 (2012).

62. Delorenzo, D. M., Bethea, D. M. & Carlson, J. K. An assessment of the diet and trophic level of Atlantic sharpnose shark *Rhizoprionodon terraenovae*. *J. Fish Biol.* **86**, 385–391 (2015).

63. Randall, J. E. *Guide to Hawaiian Reef Fishes*. (1985).

64. Powell, A. B. & Schwartz, F. J. Food of *Paralichthys dentatus* and *P. lethostigma* (Pisces: Bothidae) in North Carolina Estuaries. *Estuaries* **2**, 276 (1979).

65. Harding, J. M. & Mann, R. Diet and habitat use by bluefish, *Pomatomus saltatrix*, in a Chesapeake Bay estuary. *Environ. Biol. Fishes* **60**, 401–409 (2001).

66. Naughton, S. P. & Saloman, C. H. *Food of gag (*Mycteroperca microlepis*) from North Carolina and three areas of Florida*. (1985). doi:10.1360/zd-2013-43-6-1064

67. Matheson, R. H., Huntsman, G. R. & Manooch, C. S. Age, growth, mortality, food and reproduction of the scamp, *Mycteroperca phenax*, collected off North Carolina and South Carolina. *Bull. Mar. Sci.* **38**, 300–312 (1986).

68. Schmidt, T. W. Food habits, length-weight relationship and condition factor of young great barracuda, *Syphraena barracuda* (Walbaum), from Florida Bay, Everglades National Park, Florida. *Bull. Mar. Sci.* **44**, 163–170 (1989).

69. Grimes, C. B. Diet and feeding ecology of the vermilion snapper, *Rhomboplites aurorubens* (Cuvier) from North Carolina and South Carolina waters. *Bull. Mar. Sci.* **29**, 53–61 (1979).

70. Feitosa, J. L. L., Concentino, A. M., Teixeira, S. F. & Ferreira, B. P. Food resource use by two territorial damselfish (Pomacentridae: Stegastes) on South-Western Atlantic algal-dominated reefs. *J. Sea Res.* **70**, 42–49 (2012).

71. Cardozo, A. L. P. *et al.* Feeding ecology and ingestion of plastic fragments by *Priacanthus arenatus*: What’s the fisheries contribution to the problem? *Mar. Pollut. Bull.* **130**, 19–27 (2018).

72. Lindquist, D. & Clavijo, I. Quantifying deep reef fishes from a submersible and notes on a live collection and diet of the red barbier, *Hemanthias vivanus*. *J. Elisha Mitchell Sci. Soc.* **3**, 135–140 (1993).

73. Nelson, R. S. The life history, ecology, and population dynamics of four sympatric reef predators (*Rhomboplites aurorubens*, *Lutjanus campechanus*, Lutjanidae; *Haemulon melanurum*, Haemulidae; and *Pagrus pagrus*, Sparidae) on the east and west F. (North Carolina State University, 1988).
